# Bayesian-Inspired Dynamic-Lag Causal Graphs and Role-Aware Transformers for Landslide Displacement Forecasting

**DOI:** 10.3390/e28010007

**Published:** 2025-12-20

**Authors:** Fan Zhang, Yuanfa Ji, Xiaoming Liu, Siyuan Liu, Zhang Lu, Xiyan Sun, Shuai Ren, Xizi Jia

**Affiliations:** 1 School of Information and Communication, Guilin University of Electronic Technology, Guilin 541004, China; 21021101008@mails.guet.edu.cn (F.Z.); jiyuanfa@163.com (Y.J.); 18907830034@163.com (X.J.); 2 School of Computer Science and Engineering, Guilin University of Aerospace Technology, Guilin 541004, China; 3 Guangxi Zhuang Autonomous Region Geological Environment Monitoring Station, Nanning 530201, China; 15277405178@163.com; 4 School of Computer Science and Information Security, Guilin University of Electronic Technology, Guilin 541004, China; 21031102009@mails.guet.edu.cn; 5 School of Computer Science, Northwestern Polytechnical University, Xi’an 710072, China; zhanglu@nwpu.edu.cn; 6 Faculty of Electrical and Electronics Engineering Technology, Universiti Malaysia Pahang Al-Sultan Abdullah, Pekan 26600, Malaysia; rs0066good@gmail.com

**Keywords:** causal discovery, dynamic Bayesian networks, DAG, landslide displacement forecasting, rainfall-induced landslides

## Abstract

Increasingly frequent intense rainfall is increasing landslide occurrence and risk. In southern China in particular, steep slopes and thin residual soils produce frequent landslide events with pronounced spatial heterogeneity. Therefore, displacement prediction methods that function across sites and deformation regimes in similar settings are essential for early warning. Most existing approaches adopt a multistage pipeline that decomposes, predicts, and recombines, often leading to complex architectures with weak cross-domain transfer and limited adaptability. To address these limitations, we present CRAFormer, a causal role-aware Transformer guided by a dynamic-lag Bayesian network-style causal graph learned from historical observations. In our system, the discovered directed acyclic graph (DAG) partitions drivers into five causal roles and induces role-specific, non-anticipative masks for lightweight branch encoders, while a context-aware Top-2 gate sparsely fuses the branch outputs, yielding sample-wise attributions. To safely exploit exogenous rainfall forecasts, next-day rainfall is entered exclusively through an ICS tail with a leakage-free block mask, a non-negative readout, and a rainfall monotonicity regularizer. In this study, we curate two long-term GNSS datasets from Guangxi (LaMenTun and BaYiTun) that capture slow creep and step-like motions during extreme rainfall. Under identical inputs and a unified protocol, CRAFormer reduces the MAE and RMSE by 59–79% across stations relative to the strongest baseline, and it lowers magnitude errors near turning points and step events, demonstrating robust performance for two contrasting landslides within a shared regional setting. Ablations confirm the contributions of the DBN-style causal masks, the leakage-free ICS tail, and the monotonicity prior. These results highlight a practical path from causal discovery to forecast-compatible neural predictors for rainfall-induced landslides.

## 1. Introduction

Climate change is increasing the frequency and intensity of extreme rainfall events [1,2], and rainfall-triggered landslides remain a persistent threat to lives and infrastructure worldwide [3]. In China, recent studies have reported increasing flood risk in major river basins due to the warming climate [4], highlighting the broader impact of hydrometeorological extremes on slope and infrastructure safety. Much of southern China has a subtropical monsoon climate with uneven seasonal rainfall, and the concentrated, high-intensity precipitation during the flood season drives frequent failures. In Guangxi, a region representative of this setting, widespread hillslopes and karst, together with thin residual soils and steep gradients, create high susceptibility. Under intense rainfall, the slopes respond rapidly, shallow soils lose stability, and landslide activity becomes highly heterogeneous, with GNSS displacement records showing that single landslides often exhibit step-like offsets and progressive creep. These deformation characteristics pose severe challenges to accurate landslide displacement prediction. Consequently, developing a method that is reliable and robust, with good transferability across sites and deformation regimes in similar climatic and geomorphological settings, is critical for early warning and risk mitigation.

Most existing models optimize correlations rather than causal mechanisms, resulting in several different problems. For example, signal decomposition pipelines coupled with deep networks can fit non-linear, multiscale patterns [5], but they are complex and brittle. Multilevel decompositions (e.g., VMD, EMD) and extensive hyperparameter tuning propagate errors and impair transferability [6,7,8,9]. A central limitation concerns the treatment of rainfall forcing, where rainfall is commonly reduced to synchronous inputs or fixed-window accumulations [10]. Such summaries obscure lagged and filtered responses governed by infiltration, seepage, and pore water pressure dynamics and underutilize short-term forecasts, which are critical during extremes.

Causal discovery provides a principled alternative. For example, a Bayesian network (BN) can encode a DAG and conditional dependencies. For landslides, it can represent pathways from rainfall to displacement through hydrologic and mechanical states. Structure learning clarifies parent–child and ancestor relations, supports *d*-separation-based reasoning, and improves interpretability in confounding and non-stationary scenarios. Embedding such structures into learning can stabilize predictions during intense rainfall and alternating wetting–drying cycles [11].

In this paper, we introduce CRAFormer, a causal, role-aware end-to-end framework for landslide displacement forecasting without prior signal decomposition. At each monitoring station, CRAFormer learns a station-specific dynamic-lag causal graph (DLCG) from historical observations on a time-unrolled DAG and uses it to define role masks for that station. It then partitions the drivers into five BN-consistent roles and encodes each with a lightweight, role-masked branch. Temporal masks enforce non-anticipative constraints. A context-aware Top-2 gate fuses the branch outputs and yields sample-wise attributions, and this gated fusion allows the model to shift its reliance between self-history and hydrometeorological drivers across different deformation regimes and local geological conditions. The design links BN-style structure learning to neural parameterization and preserves clear causal semantics.

The main contributions of this paper are as follows:

1. A causally informed forecasting framework: We couple a BN-consistent structure with neural predictors to model pathways from rainfall to displacement. This approach eliminates reliance on prior signal decomposition and improves interpretability.
2. A dynamic-lag causal graph (DLCG): We perform structure learning on a time-unrolled DAG, partition the inputs into five causal roles, and encode each with role masks that operationalize *d*-separation and non-anticipativity. A context-aware Top-2 gate improves accuracy and provides sample-wise attributions.
3. A forecast-compatible rainfall pathway: Next-day rainfall enters an indirect-cause branch through a leakage-free channel with a monotone readout, which preserves non-anticipativity and stabilizes responses under strong hydrologic forcing. Numerical forecasts are used when available; otherwise, the observed $R_{t+1}$ serves as a practical proxy.
4. Evaluation on two rainfall-triggered landslides in Guangxi (LaMenTun and BaYiTun): CRAFormer improves accuracy, stability, and physical consistency at both sites under real-world conditions, especially during rainfall extremes, while retaining interpretability via role masks and gating. These experiments document the framework’s robustness across two contrasting deformation regimes within a shared regional setting.

## 2. Related Works

Landslide displacement prediction approaches are commonly grouped into three categories: physical, statistical, and data-driven. Physical models, grounded in groundwater hydrodynamics and rock–soil constitutive laws, provide strong mechanistic interpretability [12] and can resolve slope-scale soil–water migration and its control on shallow stability under rainfall forcing [13]. However, parameter identification is difficult and simulations are computationally expensive, which limits real-time use and scalability [12]. Classical statistical models, including linear regression and time-series methods, are simple and effective at short horizons but struggle with non-linear dynamics and rainfall-induced lags [14].

Deep learning has improved predictive accuracy in landslide displacement forecasting. For example, recurrent networks (e.g., LSTM and GRU) and temporal convolutional networks capture non-linear dynamics and both long- and short-term dependencies [15,16,17]. Transformer-based architectures offer attention mechanisms that assign time-varying relevance [18,19]. Spatio-temporal deep learning models with explicitly interpretable features have also been proposed for landslide displacement prediction [20]. However, three challenges remain: the need for large labeled datasets and substantial computation; limited interpretability due to opaque internal states; and weak cross-site generalization under diverse rainfall regimes and human influences [21,22].

To represent multiscale features, many studies adopt a decomposition–reconstruction pipeline, where methods such as wavelet transforms, moving averages, VMD, and EMD are used to split displacement into submodes that are modeled and then recombined [8,23,24]. Combining signal processing with learning improves the representation of multiscale structures [9,25,26], but feature selection nevertheless often relies on correlation-based metrics, such as Pearson’s correlation coefficient, the MIC, SHAP values, and elastic net regularization [15,27,28,29,30,31]. Correlation does not establish causation and can overlook coupling among drivers, which reduces transferability and robustness [32,33].

Recent studies have aimed to improve interpretability and physical consistency by combining attention mechanisms with physics-based constraints, for example, by embedding geological priors into architectures, establishing a rainfall and water level→displacement causal chain [34,35], designing deformation mechanism-assisted deep architectures for step-like reservoir landslide displacement [36], pairing decomposition with Granger causality tests to screen drivers [37], and integrating frequency-domain causal analysis, Shapley value attribution, and physics-informed structures [38,39]. These designs provide insight but face three issues: First, attention often highlights associations rather than causal effects. Second, soft physical constraints can drift during training and destabilize models. Third, added architectural complexity increases maintenance and deployment costs.

In summary, current approaches lack explicit causal modeling, faithful representation of rainfall processes across timescales, and lightweight designs that generalize across heterogeneous settings. These gaps motivated our creation of a role-aware, physics-guided framework that captures dynamic lags, uses rainfall forecasts in a leakage-free manner, and remains efficient for operational point forecasting.

## 3. Methodology

**Figure 1:**
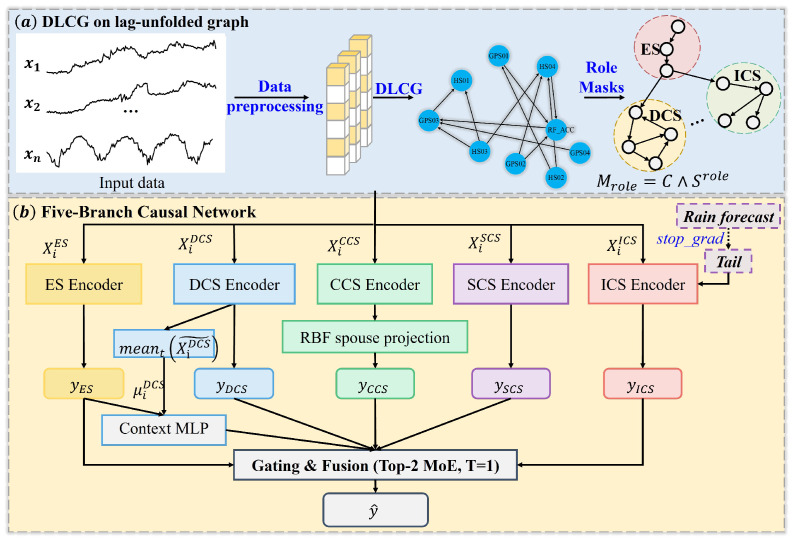
Architecture of CRAFormer with role-masked branches and gated fusion. (**a**) DLCG on lag-unfolded graph; (**b**) Five-Branch Causal Network. Five causal branches: ES (yellow, self-feedback), DCS (blue, direct causes), CCS (green, co-causes), SCS (purple, structurally irrelevant variables), and ICS (red, indirect causes).

We designed CRAFormer to decouple structure discovery from exogenous conditioning by formulating displacement forecasting with a time-unrolled Bayesian network (DBN) and a role-masked predictor. As shown in Figure 1, CRAFormer (i) performs dynamic-lag causal discovery on historical variables to obtain a DAG $\hat{G}$; (ii) derives a BN-consistent role partition via (d)-separation; (iii) encodes each role using non-anticipative temporal and structural masks; and (iv) fuses the role representations through a context-aware Top-2 gate that yields sample-wise attributions. Prediction at time t+1 uses the history together with an exogenous rainfall channel $R_{t+1}$ that is excluded from discovery and injected through a leakage-free tail, preventing information leakage from the forecast horizon while staying compatible with operational rainfall forecasts. The historical variables are partitioned into five disjoint roles, self-feedback (ES), direct causes (DCSs), co-causes (CCSs), indirect causes (ICSs), and structurally irrelevant variables (SCSs), with each processed by its role-masked encoder.

The ICS branch ingests the exogenous next-day rainfall $R_{t+1}$ through a leakage-free tail token. The tail attends to history, whereas history cannot attend to the tail, which preserves non-anticipativity. We also apply a lightweight, regime-selective monotonicity prior that softly encourages a non-decreasing displacement response to effective rainfall during high- or rising-rainfall episodes where pore pressure-driven acceleration is expected to dominate the short-horizon dynamics. Outside these regimes, the prior is inactive. A compact routing context derived from ES and DCS summaries feeds a temperature-scaled Top-2 gate, which selects the two most informative role branches and forms a convex combination of their scalar outputs. The normalized gate weights provide sample-wise attributions.

### 3.1. Dynamic-Lag Causal Discovery in a Time-Unrolled Bayesian Network

We adopt the DLCG as the structural prior for CRAFormer. Concretely, we learn a DAG over lag-expanded historical nodes in a daily, time-unrolled dynamic BN and aggregate multiple runs using a stationary bootstrap to obtain a robust directed graph $\hat{G}$. In line with standard time-series causal discovery, we interpret $\hat{G}$ as a statistically supported summary of recurrent lagged dependencies rather than a fully specified physical model, and its causal reading relies on four working assumptions: (i) acyclicity of the time-unrolled graph; (ii) causal sufficiency for the observed driver set; (iii) the approximate stationarity of the short-lag dependence structure over the study period, rather than the strict stationarity of the raw displacement levels; and (iv) the Markov and faithfulness conditions [40]. When these assumptions are only approximately satisfied, the main effect is a loss of power. Weak or regime-specific dependencies are more likely to be omitted and some indirect or confounded links may remain, while BY–FDR control and directional thresholding keep $\hat{G}$ relatively sparse. We therefore use $\hat{G}$ as a conservative structural prior for role masking in CRAFormer rather than as a fully identifiable physical model.

The search space consists solely of historical lagged nodes at daily resolution:

(1) $$V_{0}=\left\{\;V_{j}^{\left(t-\tau \right)}\;|\;V_{j}\in \left\{X_{\mathrm{disp}}\right\}\cup {\left\{X_{j}\right\}}_{j\in I_{\mathrm{drv}}},\;\tau =0,\ldots ,L-1\right\}.$$

where $I_{\mathrm{drv}}$ denotes the index set of environmental drivers; j∈I indexes the variables with $X_{\mathrm{disp}}$ being the displacement series; *t* is the last observed day; τ∈{0,…,L−1} is the lag; and *L* is the look-back window.

To prevent anticipative edges, we exclude future rainfall nodes within the prediction horizon from $V_{0}$ and later treat them as exogenous inputs in the ICS tail (gauge oracle $R_{\mathrm{gau}}$; see Section 3.3.1) [41,42]. Candidate parents must strictly precede each node in time:

(2) $$P\;\left(X_{j}^{\left(t-\tau \right)}\right)\subseteq \left\{\;X_{k}^{\left(t-\tau^{\prime }\right)}:\tau^{\prime }>\tau ,\;k\in I\;\right\}.$$

where P(·) is the candidate–parent set; j,k∈I; *t* is the last observed day; and $\tau ,\tau^{\prime }\in \left\{0,\ldots ,L-1\right\}$ are lags with $\tau^{\prime }>\tau$.

We impose hard constraints that forbid edges from displacement to drivers, and we use domain priors only to break ties [43,44]. *B* stationary bootstrap resamples are drawn with an expected block length of 7 days, and a constraint-based skeleton is learned for each resample [45]. A sensitivity analysis further shows that the learned DLCG remains stable for block lengths b∈{3,7,14} days (see Appendix B.4, Figure A9). Conditional independence is tested primarily with a kernel-based test (HSIC/KCI) [46,47]. *p*-values are computed by block permutation with a 7-day block to respect serial dependence. Within each conditioning order, multiplicity is controlled using the Benjamini–Yekutieli FDR (BY-FDR) at α=0.05 [48]. For low-order conditioning sets (|Z|≤2) that pass linearity and normality diagnostics, we fall back to the Gaussian partial correlation test (Fisher–*z*):

(3) $$z\;=\;\frac{1}{2}\sqrt{\;n-|Z|-3\;}\;\ln\;\frac{1+r_{XY\;\cdot \;Z}}{1-r_{XY\;\cdot \;Z}},\;p\;=\;2\;\Phi \;\left(-|z|\right),$$

where $r_{XY\;\cdot \;Z}$ is the partial correlation and Φ is the standard normal CDF. To account for serial dependence, we calibrate *p*-values for the Fisher–*z* test using the same 7-day block permutation scheme rather than relying on the i.i.d. normal approximation.

The pruned skeleton is oriented using v-structures and Meek rules under hard temporal constraints. We also extract the collider and spouse sets (C,S) for the displacement target, to depth $L_{\mathrm{col}}$, to guide downstream CCS masking. Directed edges are aggregated across bootstrap resamples, and we keep u→v in $\hat{G}$ if

(4) $$L_{\to }\;\geq \;0.60\;\mathrm{and}\;L_{\to }-U_{\leftarrow }\;\geq \;0.30,$$

where $L_{\to }$ and $U_{\leftarrow }$ are the Clopper–Pearson lower and upper confidence bounds for the forward and reverse selection rates, respectively [49]. We treat these directional selection rates as approximate posterior inclusion probabilities and choose $\left(L_{\to },\Delta \right)=\left(0.60,0.30\right)$ as a compromise between retaining sufficiently many edges and discarding unstable orientations. Table A1 in Appendix A.1 further reports a small threshold sensitivity study showing that the aggregated DLCG and the DCS/ICS role masks for the displacement node are stable under moderate variations of $\left(L_{\to },\Delta \right)$. The resulting $\hat{G}$ and (C,S) serve as priors for role partitioning. Full pseudocode and hyperparameters are provided in Algorithm 1 and Table A3.
**Algorithm 1** DLCG: dynamic-lag causal graph on $V_{0}$**Require:** time series $X\;\in \;R^{T\times D}$, lookback *L*, drivers $I_{\mathrm{drv}}$, priors $\left(E^{+},E^{-}\right)$, BY level α,      bootstraps *B*, block length, collider depth $L_{\mathrm{col}}$, target**Ensure:** robust graph $\hat{G}=\left(V_{0},E\right)$, collider set C, spouse set S  1:$V_{0}\leftarrow \left\{V_{j}^{\left(t-\tau \right)}:j\in \left\{\mathrm{disp}\right\}\cup I_{\mathrm{drv}},\;\tau =0,\ldots ,L-1\right\}$   ▹ exclude forecasts  2:Build temporal mask $M\left[u,v\right]\leftarrow \left(\mathrm{lag}\left(u\right)>\mathrm{lag}\left(v\right)\right)\wedge \left(\left(u\to v\right)\notin E^{-}\right)$  3:**for** b=1 to *B* **do**                ▹ stationary bootstrap  4:     $X_{b}\leftarrow S\mathrm{TATIONARY}B\mathrm{OOTSTRAP}\left(X,\mathrm{block}\;\mathrm{length}\right)$  5:     $\left(G_{b},C_{b},S_{b}\right)\leftarrow L\mathrm{EARN}O\mathrm{NCE}\left(X_{b},V_{0},M,E^{+},E^{-},\alpha ,L_{\mathrm{col}},\mathrm{target}\right)$  6:**end for**  7:Aggregate $\left\{G_{b}\right\}$ into directed edges *E* using Clopper–Pearson directional bounds; keep u→v if $L_{\to }\;\geq \;0.60$ and $L_{\to }\;-\;U_{\leftarrow }\;\geq \;0.30$  8:$\hat{G}\leftarrow \left(V_{0},E\right)$; $C\leftarrow {⋃}_{b}C_{b}$; $S\leftarrow {⋃}_{b}S_{b}$  9:**return**$\;(\hat{G},C,S)$10:**procedure **$L\mathrm{EARN}O\mathrm{NCE}(X_{b},V_{0},M,E^{+},E^{-},\alpha ,L_{\mathrm{col}},\mathrm{target}$)11:     Initialize undirected skeleton *G* under mask *M*12:     **for** k=0 to $k_{\max}$ **do**       ▹ size-adaptive conditioning order13:         Test conditional independence (HSIC/KCI; Fisher–*z* fallback under |Z|≤2 and diagnostics passed) and prune order-*k* edges with BY–FDR control14:     **end for**15:     Orient *G* using *v*-structures and Meek rules; apply $E^{+}$ only for tie-breaking; enforce $E^{-}$16:     $\left(C,S\right)\leftarrow M\mathrm{INE}C\mathrm{OLLIDER}S\mathrm{POUSE}\left(G,\mathrm{target},L_{\mathrm{col}}\right)$17:     **return** (G,C,S)18:**end procedure**

### 3.2. Causal Role Partitioning

Given the DLCG $\hat{G}$, we derive the following role masks as the algebraic image of *d*-separation on the time-unrolled BN: ES, DCSs, CCSs, ICSs, and SCSs. These masks constitute the visibility sets used by the encoders: ES contains temporal self-lags of the target; DCSs equal the parent set ${\mathrm{Pa}}_{\hat{G}}\;\left(X_{\mathrm{disp}}^{\left(t+1\right)}\right)$ with ES lags removed; ICSs collect proper ancestors and mediators, that is, ${\mathrm{An}}_{\hat{G}}\;\left(X_{\mathrm{disp}}^{\left(t+1\right)}\right)\setminus \left(\mathrm{ES}\cup \mathrm{DCS}\right)$; CCSs gather *v*-structure colliders on ancestral paths to the target and their spouses; SCSs contain all remaining historical nodes, that is, the complement of (ES∪DCS∪ICS∪CCS).

ES comprises the contemporaneous target value and its previous L−1 lags as follows:

(5) $$\mathrm{ES}\;=\;\left\{\;X_{\mathrm{disp}}^{\left(t\right)},\;X_{\mathrm{disp}}^{\left(t-1\right)},\;\ldots ,\;X_{\mathrm{disp}}^{\left(t-L+1\right)}\;\right\},$$

where $X_{\mathrm{disp}}$ is the displacement series, *t* the last observed day, and *L* the look-back window (days).

DCSs comprise the one-hop parents of the target at time t+1, excluding ES:

(6) $${\mathrm{DCS}}_{i}=\left\{\;V_{j}^{\left(t-\tau \right)}\;|\;V_{j}^{\left(t-\tau \right)}\to X_{i}^{\left(t+1\right)}\in \hat{G}\right\}\setminus {\mathrm{ES}}_{i},$$

where *i* indexes the target ($X_{i}\;\equiv \;X_{\mathrm{disp}}$); $V_{j}\in \left\{\mathrm{disp}\right\}\cup I_{\mathrm{drv}}$; τ∈{0,…,L−1} is the lag; → denotes a directed edge in $\hat{G}$; and ${\mathrm{ES}}_{i}$ is the ES set for *i*.

CCSs comprise collider nodes and their spouses:

(7) $$\begin{array}{c} C_{i}=\left\{\;c\in V_{0}\;|\;\exists \;u\neq v:\;u\to c\leftarrow v,\;u\in {\mathrm{An}}_{\hat{G}}\;\left(X_{i}^{\left(t+1\right)}\right)\right\},\\ S_{i}=\left\{\;s\in V_{0}\;|\;\exists \;c\in C_{i}:\;s\to c\right\},\\ {\mathrm{CCS}}_{i}=\left(C_{i}\cup S_{i}\right)\setminus \left({\mathrm{ES}}_{i}\cup {\mathrm{DCS}}_{i}\right), \end{array}$$

where ${\mathrm{An}}_{\hat{G}}\left(\;\cdot \;\right)$ denotes the set of ancestors in $\hat{G}$. A collider has two incoming edges that form a v-structure, and spouses are nodes that share the same collider as a child. We apply the spouse-informed kernel projection only when the spouse index set is non-empty; otherwise, it is the identity $\Psi_{i}=Z_{i}$.

ICSs comprise intermediate nodes that influence the next-day target through directed paths with a length of at least 2, excluding ES, DCSs, and CCSs:

(8) $${\mathrm{ICS}}_{i}\;=\;\left\{\;V_{j}^{\left(t-\tau \right)}\;|\;\exists \;\gamma \in {\mathrm{Paths}}_{\hat{G}}\;\left(V_{j}^{\left(t-\tau \right)},\;X_{i}^{\left(t+1\right)}\right)\;\mathrm{with}\;\left|\gamma \right|\geq 2\right\}\setminus \left({\mathrm{ES}}_{i}\cup {\mathrm{DCS}}_{i}\cup {\mathrm{CCS}}_{i}\right).$$

where $V_{j}\in \left\{\mathrm{disp}\right\}\cup I_{\mathrm{drv}}$; *i* indexes the displacement target ($X_{i}\;\equiv \;X_{\mathrm{disp}}$); *t* is the last observed day; τ∈{0,…,L−1} is the lag; ${\mathrm{Paths}}_{\hat{G}}\left(u,v\right)$ are directed paths in $\hat{G}$; |γ| is the path length (edges); and ${\mathrm{ES}}_{i},{\mathrm{DCS}}_{i},{\mathrm{CCS}}_{i}$ are as defined above.

SCSs comprise all remaining nodes:

(9) $${\mathrm{SCS}}_{i}\;=\;V_{0}\setminus \left({\mathrm{ES}}_{i}\cup {\mathrm{DCS}}_{i}\cup {\mathrm{CCS}}_{i}\cup {\mathrm{ICS}}_{i}\right).$$

We assign roles in the following precedence order:

(10) ES≻DCS≻CCS≻ICS≻SCS.

This order ensures that the five sets are pair-wise disjoint and exhaustive over $V_{0}$, and we break ties due to sampling variability in this order. Because $\hat{G}$ is obtained by aggregating stationary bootstrap graphs under conservative directional thresholds (Section 3.1), the resulting ES/DCS/ICS/CCS/SCS masks inherit a degree of robustness to sampling variability. For the displacement node, we verify on representative stations that the dominant short-lag hydrologic lags assigned to DCSs and ICSs remain in the same roles when the DLCG is recomputed on bootstrap subsets, with only a few marginal lags switching between ICSs and SCSs (Appendix A.2, Table A2). This stability limits the impact of structural uncertainty on the Top-2 gating and supports the use of the five-way partition as a reliable basis for interpretability.

### 3.3. Multibranch Displacement Prediction Model

**Figure 2:**
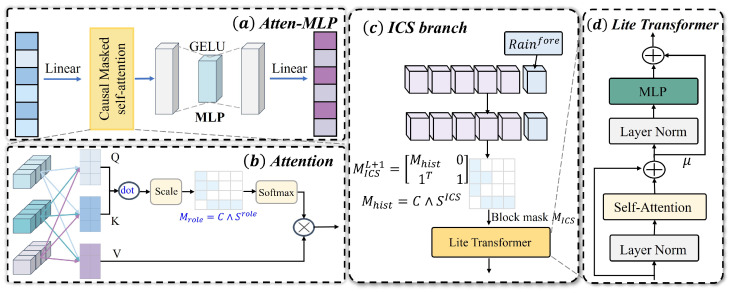
Role-masked branches and lightweight encoders in CRAFormer: (**a**) Attention–MLP encoder: linear projection, causal/masked self-attention, GELU-MLP, and linear head. (**b**) Attention with role mask $M_{\mathrm{role}}=C\wedge S^{\left(\mathrm{role}\right)}$. (**c**) ICS branch: an *exogenous next-day rainfall* token $R_{\mathrm{gau}}$ (gauge oracle) is appended as a tail; the block mask allows the tail to read history but not vice versa. (**d**) Lite Transformer cell with pre-norm, residual mixing μ, and a compact MLP.

Guided by the role partitioning, CRAFormer adopts a multibranch architecture. Each causal subset is encoded with temporal and structural masks (Figure 2), and this separation isolates distinct sources of influence and enforces non-anticipativity, where C is a strictly lower-triangular temporal mask and $S^{\left(\mathrm{role}\right)}$ is the role-specific visibility mask derived from $\hat{G}$. Each branch applies masked self-attention with the following composite mask:

(11) $$M_{\mathrm{role}}=C\wedge S^{\left(\mathrm{role}\right)},$$

where ∧ denotes the element-wise logical AND. The masking operator Ω(·) adds 0 to visible entries and −∞ to masked entries before applying softmax to the attention logits.

Role-generic encoder (ES/DCS/SCS/CCS): Roles r∈{ES,DCS,SCS,CCS} share the encoder shown in Figure 2a, where inputs are linearly projected and positionally encoded. We then apply single-head causal self-attention with the mask $M_{r}=C\wedge S^{\left(r\right)}$ [50], followed by a small MLP and a linear head:

(12) $${\tilde{X}}_{i}^{\left(r\right)}=\mathrm{PE}\;\left(X_{i}^{\left(r\right)}W_{\mathrm{in}}^{\left(r\right)}\right)\in R^{L\times d},$$

where *i* indexes samples r∈{ES,DCS,SCS,CCS}; $X_{i}^{\left(r\right)}$ is the length-*L* input for role *r*; PE(·) is the positional encoding and *d* the hidden width.

(13) $$Q^{\left(r\right)}={\tilde{X}}_{i}^{\left(r\right)}W_{q}^{\left(r\right)},\;K^{\left(r\right)}={\tilde{X}}_{i}^{\left(r\right)}W_{k}^{\left(r\right)},\;V^{\left(r\right)}={\tilde{X}}_{i}^{\left(r\right)}W_{v}^{\left(r\right)},$$

where $W_{\mathrm{in}}^{\left(r\right)},W_{q,k,v}^{\left(r\right)}$ are learned parameters.

(14) $$A_{i}^{\left(r\right)}=\mathrm{softmax}\;\left(\frac{Q^{\left(r\right)}K^{(r)\;⊤}}{\sqrt{d}}+\Omega \left(M_{r}\right)\right),$$

where $\Omega (M_{r})$ applies the binary mask $M_{r}=C\wedge S^{\left(r\right)}$ (with C being strictly causal and $S^{\left(r\right)}$ role visibility).

(15) $$Z_{i}^{\left(r\right)}=A_{i}^{\left(r\right)}V^{\left(r\right)}\in R^{L\times d},\;z_{i}^{\left(r\right)}=Z_{i}^{\left(r\right)}\left[L,:\right]\in R^{d},$$

where $Z_{i}^{\left(r\right)}$ are token features and $z_{i}^{\left(r\right)}=Z_{i}^{\left(r\right)}\left[L,:\right]$ the last token.

(16) $$y_{i}^{\left(r\right)}=w_{2}^{(r)\;⊤}\;\mathrm{GELU}\;\left(z_{i}^{\left(r\right)}W_{1}^{\left(r\right)}\right)+b_{r}.$$

where $W_{1}^{\left(r\right)},w_{2}^{\left(r\right)},b_{r}$ are learned parameters; and $y_{i}^{\left(r\right)}$ is the scalar head.

For ES, DCSs, and SCSs, we use a scalar head $y_{i}^{\left(r\right)}$, and for CCSs, we also retain the token map $Z_{i}^{\left(\mathrm{CCS}\right)}$ for downstream projection. The encoder is low-capacity and uses single-head attention, a small hidden dimension *d*, dropout 0.1, and $L_{2}$ weight decay of $10^{-4}$.

In the CCS branch, let $Z_{i}\equiv Z_{i}^{\left(\mathrm{CCS}\right)}$ from (12) to (16). Given the spouse-token index set $I_{S}$, we remove components explained by spouses using an RBF kernel projection as follows:

(17) $$Z_{i,S}=Z_{i}\left[I_{S},:\right],\;K_{SS}=\kappa \left(Z_{i,S},Z_{i,S}\right),\;K_{TS}=\kappa \left(Z_{i},Z_{i,S}\right),$$

where $\kappa \left(u,v\right)=\exp\;\left({-∥u-v∥}_{2}^{2}/\left(2\sigma^{2}\right)\right)$ is the RBF kernel; ε>0 stabilizes the inverse.

(18) $${\hat{Z}}_{i|S}=K_{TS}{\left(K_{SS}+\varepsilon I\right)}^{-1}Z_{i,S},\;\Psi_{i}=Z_{i}-{\hat{Z}}_{i|S},$$

where I is the identity; and vec(·) vectorizes a matrix.

(19) $$y_{i}^{\mathrm{CCS}}=w_{\mathrm{CCS}}^{⊤}\mathrm{vec}\left(\Psi_{i}\right)+b_{\mathrm{CCS}},$$

If $|I_{S}|=0$, set $\Psi_{i}=Z_{i}$.

ICS branch encoder: The ICS branch appends the gauge oracle exogenous next-day rainfall $R_{\mathrm{gau}}$ as the tail token shown in Figure 2c, which is not an NWP forecast. We construct an effective rainfall driver from the rainfall history and $R_{t+1}$. When a numerical forecast of $R_{t+1}$ is available, we use it; otherwise, the observed $R_{t+1}$ serves as a practical proxy at evaluation time. We pass $R_{t+1}$ through a detach operation to stop gradients before fusion.

(20) $${\tilde{X}}_{i}^{\mathrm{ICS}}=\mathrm{PE}\;\left(X_{i}^{\mathrm{ICS}}W_{\mathrm{in}}^{\mathrm{ICS}}\right)\in R^{L\times d}.$$

(21) $$r_{\mathrm{eff},i}=\mathrm{ReLU}\;\left(a^{⊤}R_{i}+v^{⊤}\phi \left(WR_{i}+b\right)+\beta ,\;\mathrm{detach}\;\left(R_{\mathrm{gau},i}\right)-\theta \right).$$

where *i* indexes samples; $X_{i}^{\mathrm{ICS}}$ is the length-*L* ICS input, PE(·) positional encoding, *d* the hidden width, and $W_{\mathrm{in}}^{\mathrm{ICS}}$ a learned projection; $R_{i}$ are rainfall features (history + $R_{t+1}$); $R_{\mathrm{gau},i}$ is the gauge oracle next-day rainfall; detach(·) blocks gradients; ϕ(·) is a point-wise non-linearity; a,v,W,b,β,θ are learned parameters; and ReLU(x,y):=max(x,y).

We map the effective rainfall score $r_{\mathrm{eff},i}$ to a non-negative tail token representing the gauge oracle exogenous next-day rainfall input, and we append this token to the sequence

(22) $$x_{\mathrm{tail},i}=W_{r}\;r_{\mathrm{eff},i},\;W_{r}=\mathrm{softplus}\left({\tilde{W}}_{r}\right)⪰0,$$

(23) $$Z_{\mathrm{in},i}^{\mathrm{ICS}}=\left[\begin{array}{c} {\tilde{X}}_{i}^{\mathrm{ICS}}\\ x_{\mathrm{tail},i} \end{array}\right]\in R^{(L+1)\times d}.$$

where $r_{\mathrm{eff},i}$ is as above; ${\tilde{W}}_{r}$ is unconstrained and $W_{r}=\mathrm{softplus}\left({\tilde{W}}_{r}\right)$ (element-wise) so $W_{r}⪰0$; $x_{\mathrm{tail},i}\in R^{d}$ is the non-negative tail token; ${\tilde{X}}_{i}^{\mathrm{ICS}}\in R^{L\times d}$; [·;·] stacks along the time axis to append the tail as the (L+1)-st token, yielding $Z_{\mathrm{in},i}^{\mathrm{ICS}}\in R^{(L+1)\times d}$; and *d* is the hidden width.

When $M_{\mathrm{hist}}=C\wedge S^{\left(\mathrm{ICS}\right)}$, we apply a block causal mask that allows the tail to attend to all historical tokens while preventing attention from history to the tail, which preserves non-anticipativity:

(24) $$M_{\mathrm{ICS}}=\left[\begin{array}{cc} M_{\mathrm{hist}} & 0\\ 1_{L}^{\;⊤} & 1 \end{array}\right],$$

where $1_{L}$ is a length-*L* vector of ones.

The prediction head reads only the tail token with element-wise non-negative weights:

(25) $$y_{i}^{\mathrm{ICS}}=w_{h}^{⊤}z_{i,\mathrm{tail}}+b_{h},\;w_{h}=\mathrm{softplus}\left({\tilde{w}}_{h}\right)⪰0.$$

where softplus(·) enforces element-wise non-negativity on $W_{r}$ and $w_{h}$. We apply a detach operation to the exogenous rainfall input so that no gradients flow from the tail token back to historical tokens. However, non-negative mixing alone does not guarantee exact monotonicity because the tail token is produced by attention and MLP layers. We regularize monotonic behavior with $L_{\mathrm{mono}}^{\mathrm{rain}}$, applied only when the rainfall trigger $m_{i}=1$. Note that the intent is not to impose global monotonicity of displacement with respect to rainfall but to encode a local short-horizon prior under strongly forced, rainfall-dominated conditions.

Lite Transformer cell: Each attention block in Figure 2d adopts a pre-norm design with a learnable residual mixing coefficient $\mu_{\ell }\in \left(0,1\right)$ [51]. For input $H^{\ell }$ and mask M,

(26) $$\begin{array}{cccc} A^{\ell } & =\mathrm{Attn}\;\left(\mathrm{LN}(H^{\ell });M\right), & H^{\ell^{\prime }} & =H^{\ell }+\mu_{\ell }\;A^{\ell }, \end{array}$$

(27) $$\begin{array}{cccc} F^{\ell } & =\mathrm{MLP}\;\left(\mathrm{LN}(H^{\ell^{\prime }})\right), & H^{\ell +1} & =H^{\ell^{\prime }}+F^{\ell }, \end{array}$$

where Attn(·;M) denotes masked attention and LN denotes layer normalization. This block reduces the parameter count, stabilizes training, and aligns with panels (a)–(d).

#### 3.3.1. Context-Aware Gating and Fusion

Each attention block in Figure 2d uses a pre-norm design with a learnable residual mixing coefficient $\mu_{\ell }\in \left(0,1\right)$ [51]. Given the input $H^{\ell }$ and mask M, we compute

(28) $$\mu_{i}^{\mathrm{DCS}}={\mathrm{mean}}_{t}\;\left({\tilde{X}}_{i}^{\mathrm{DCS}}\right)\in R^{d},\;c_{i}={\mathrm{MLP}}_{\mathrm{ctx}}\;\left(\;[\;y_{i}^{\mathrm{ES}};\;\mu_{i}^{\mathrm{DCS}}\;]\;\right)\in R^{h_{\mathrm{ctx}}},$$

where ${\mathrm{mean}}_{t}$ denotes the temporal mean. The vector $c_{i}$ summarizes recent displacement and direct drivers and serves as a shared routing context [52].

Given branch-level scalar outputs ${\left\{y_{i,j}\right\}}_{j\in J}$ with J={ES,DCS,CCS,ICS,SCS}, we compute per-branch logits using an affine transform of $y_{i,j}$ and a shared context term as follows:

(29) $$z_{i,j}=a_{j}\;y_{i,j}+v^{⊤}c_{i}+b_{j},\;j\in J.$$

where *i* indexes samples; j∈J is the branch; $y_{i,j}$ is branch *j*’s scalar output; $a_{j},b_{j}$ are learned gate scalars; and $v,c_{i}\in R^{h_{\mathrm{ctx}}}$ are the shared-context weight and vector (so $z_{i,j}$ is a logit).

Soft routing probabilities with temperature τ>0 are

(30) $$\pi_{i,j}=\frac{\exp\;\left(z_{i,j}/\tau \right)}{\sum_{k\in J}\exp\;\left(z_{i,k}/\tau \right)},\;j\in J.$$

where *i* indexes samples; j,k∈J are index branches; τ>0 is the softmax temperature; the denominator sums over J; and $\left\{\pi_{i,j}\right\}$ are normalized ($\sum_{j\in J}\pi_{i,j}=1$).

We use temperature-scaled soft targets for training [53], and select the Top-2 routing probabilities and record their indices as $S_{i}=\mathrm{Top}-2\left({\left\{\pi_{i,j}\right\}}_{j\in J}\right)$. We then renormalize over $S_{i}$ for sparse MoE routing [54,55], and the normalized gates fuse the branch outputs as follows:

(31) $$g_{i,j}=\frac{\pi_{i,j}\;1\left[\;j\in S_{i}\;\right]}{\sum_{\ell \in S_{i}}\pi_{i,\ell }},\;{\hat{y}}_{i}=\sum_{j\in J}g_{i,j}\;y_{i,j},$$

where 1{·} denotes the indicator function, and $\sum_{j\in J}g_{i,j}=1$. We break ties by the fixed role order ES≻DCS≻CCS≻ICS≻SCS to ensure determinism.

To promote decisive routing and stabilize training, we penalize the entropy of the pre-truncation probability vector as follows:

(32) $$L_{\mathrm{gate}}=\lambda_{\mathrm{ent}}\sum_{i}H\left(\pi_{i}\right),\;H\left(\pi_{i}\right)=-\sum_{j\in J}\pi_{i,j}\;\log\pi_{i,j}.$$

This entropy minimization regularizer encourages confident assignments [56]. We also apply $L_{2}$ weight decay to $\left\{a_{j},b_{j},v\right\}$. In practice, a temperature τ∈[0.3,1.0] and $\lambda_{\mathrm{ent}}\in \left[10^{-3},10^{-2}\right]$ yield stable Top-2 routing without gate collapse.

#### 3.3.2. Joint Loss Function

The total loss combines prediction error, an SCS gate sparsity penalty, an optional rainfall-triggered monotonicity term, gating entropy regularization, and $L_{2}$ weight decay [56,57] as follows:

(33) $$L_{\mathrm{total}}\;=\;L_{\mathrm{pred}}\;+\;\lambda_{\mathrm{scs}}\;{\bar{g}}_{\mathrm{SCS}}\;+\;\lambda_{\mathrm{mono}}\;L_{\mathrm{mono}}^{\mathrm{rain}}\;+\;\lambda_{\mathrm{ent}}\;L_{\mathrm{gate}}\;+\;\gamma \;{∥\Theta ∥}_{2}^{2}.$$

where $\lambda_{\mathrm{scs}},\lambda_{\mathrm{mono}},\lambda_{\mathrm{ent}},\gamma \geq 0$ are weights (typical ranges: $\lambda_{\mathrm{scs}}\in \left[10^{-3},10^{-2}\right]$; $\lambda_{\mathrm{mono}}\in \left[10^{-3},10^{-1}\right]$; $\lambda_{\mathrm{ent}}\in \left[10^{-3},10^{-2}\right]$; and $\gamma \in [10^{-5},10^{-3}]$); $\lambda_{\mathrm{mono}}=0$ is set to disable the monotonicity term; ${\bar{g}}_{\mathrm{SCS}}$ denotes the batch-mean SCS gate activation; and Θ collects all trainable parameters. To encourage decisive routing, we include a small Shannon entropy penalty $L_{\mathrm{gate}}$ in the pre-truncation gate probabilities (see Section 3.3.1).

The prediction loss and the batch-mean SCS gate are

(34) $$L_{\mathrm{pred}}\;=\;\frac{1}{B}\sum_{i=1}^{B}{\left({\hat{y}}_{i}-y_{i}\right)}^{2},\;{\bar{g}}_{\mathrm{SCS}}\;=\;\frac{1}{B}\sum_{i=1}^{B}g_{i,\mathrm{SCS}}.$$

where *B* denotes the minibatch size. The target $y_{i}$ is the cumulative displacement over the prediction horizon *H*,

(35) $$y_{i}\;\equiv \;\sum_{h=1}^{H}\Delta {\mathrm{disp}}_{t_{i}+h},$$

and ${\hat{y}}_{i}$ is its prediction (both in physical units). The quantity $g_{i,\mathrm{SCS}}\in \left[0,1\right]$ is the normalized SCS gate, and $\sum_{j\in J}g_{i,j}=1$.

We activate the physics prior only under heavy or rising rainfall and only when the ICS gate is active:

(36) $$m_{i}\;=\;1\;\left[\;R_{7,i}\geq \tau_{r}\;\vee \;\left(r_{\mathrm{eff},i}-r_{\mathrm{eff},i}^{\mathrm{prev}}\right)\geq \tau_{\Delta }\;\right]\;\cdot \;1\;\left[\;g_{i,\mathrm{ICS}}\geq \tau_{g}\;\right].$$

where $R_{7,i}$ denotes the 7-day accumulated rainfall, and $r_{\mathrm{eff},i}^{\mathrm{prev}}$ is the previous step’s effective rainfall score. We set $\tau_{r}$ to the 80th percentile of $R_{7}$ on the training split, and $\tau_{\Delta }$ and $\tau_{g}$ are fixed thresholds (for example, $\tau_{\Delta }>0$ and $\tau_{g}=0.5$). These criteria restrict the monotonicity prior to episodes with already high or rapidly increasing rainfall and strong routing to the hydrologic ICS branch, which are precisely the regimes where a non-decreasing short-horizon response to additional rainfall is physically plausible. In drier periods or during post-event drainage and partial recovery, the trigger satisfies $m_{i}=0$, and the network is free to represent non-monotonic behavior such as relaxation or partial re-stabilization.

This term thus acts as a monotonicity regularizer; it penalizes negative finite-difference slopes only when $m_{i}=1$. In practice, $\lambda_{\mathrm{mono}}$ is chosen in the low range given above, so $L_{\mathrm{mono}}^{\mathrm{rain}}$ provides a soft preference rather than a hard constraint. If the data strongly support local decreases even under high rainfall, CRAFormer can accommodate them via the ES/DCS branches and the Top-2 routing.

Let $\Delta {\hat{y}}_{i}\;\equiv \;{\hat{y}}_{i}^{\left(+\right)}-{\hat{y}}_{i}$ denote the finite-difference slope with respect to a small increase in the effective rainfall driver. The penalty ${\left[\max\left(0,\;{\hat{y}}_{i}-{\hat{y}}_{i}^{\left(+\right)}\right)\right]}^{2}$ is equivalent to ${\left[\max\left(0,\;-\Delta {\hat{y}}_{i}\right)\right]}^{2}$ and follows monotonic constraint regularization in neural networks [58].

(37) $$L_{\mathrm{mono}}^{\mathrm{rain}}\;=\;\frac{\sum_{i=1}^{B}m_{i}\;{\left[\max\;\left(0,\;{\hat{y}}_{i}-{\hat{y}}_{i}^{\left(+\right)}\right)\right]}^{2}}{\sum_{i=1}^{B}m_{i}\;+\;\varepsilon }\;,$$

where ε>0 is a small constant for numerical stability.

We probe a small relative positive step in the effective rainfall driver as follows:

(38) $$r_{\mathrm{eff},i}^{\left(+\right)}\;=\;r_{\mathrm{eff},i}\;+\;\mathrm{detach}\;\left(\eta_{r}\;(\;|r_{\mathrm{eff},i}\left|+\varepsilon_{r}\;\right)\right).$$

where $\eta_{r}\in \left(0,1\right)$ (default 0.1) and $\varepsilon_{r}>0$ prevents vanishing steps near zero. We set $\tau_{r}$ to the 80th percentile of $R_{7}$ on the training split, $\tau_{\Delta }$ to the 80th percentile of Δr (the first difference of $r_{\mathrm{eff}}$ on the training split), and $\tau_{g}=0.5$. The perturbed output ${\hat{y}}_{i}^{\left(+\right)}={\hat{y}}_{i}\;\left(r_{\mathrm{eff},i}^{\left(+\right)}\right)$ is computed using a detach operation so that gradients do not flow through the step.

## 4. Study Areas and Data

**Figure 3:**
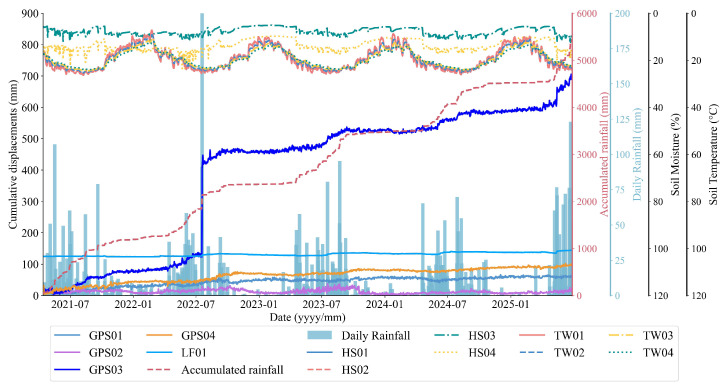
Representative time series at LaMenTun: displacement, rainfall metrics, volumetric water content, and soil temperature.

**Figure 4:**
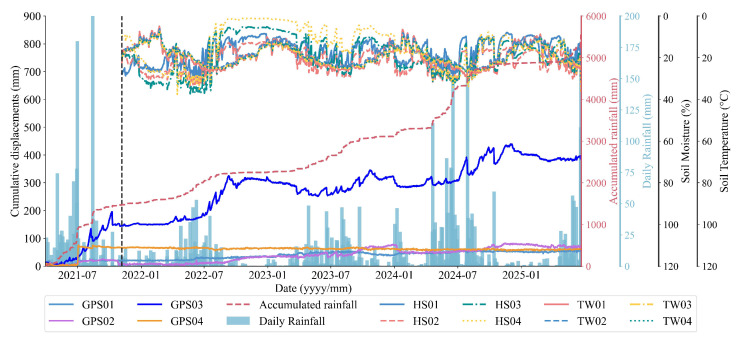
Representative time series at BaYiTun: displacement, rainfall metrics, volumetric water content, and soil temperature.

The landslides at LaMenTun and BaYiTun in Guangxi are selected as case studies. Both sites lie in humid, subtropical, monsoon-dominated settings and exhibit rainfall-triggered deformation patterns that are typical for this region of southern China; however, they differ markedly in their deformation regimes. Therefore, they offer two contrasting examples within a shared climatic and geomorphological setting. The LaMenTun landslide is characterized by progressive, seasonally modulated creep behavior with periodic acceleration and step-like responses, while the BaYiTun landslide displays more episodic, leap-frog deformation dominated by intermittent and spatially heterogeneous movement (Figure 3 and Figure 4). Both sites host continuous GNSS stations and multisensor environmental monitoring, providing long-term, high-resolution time-series data for training and validating mechanism-aware prediction models.

### 4.1. LaMenTun Landslide

**Figure 5:**
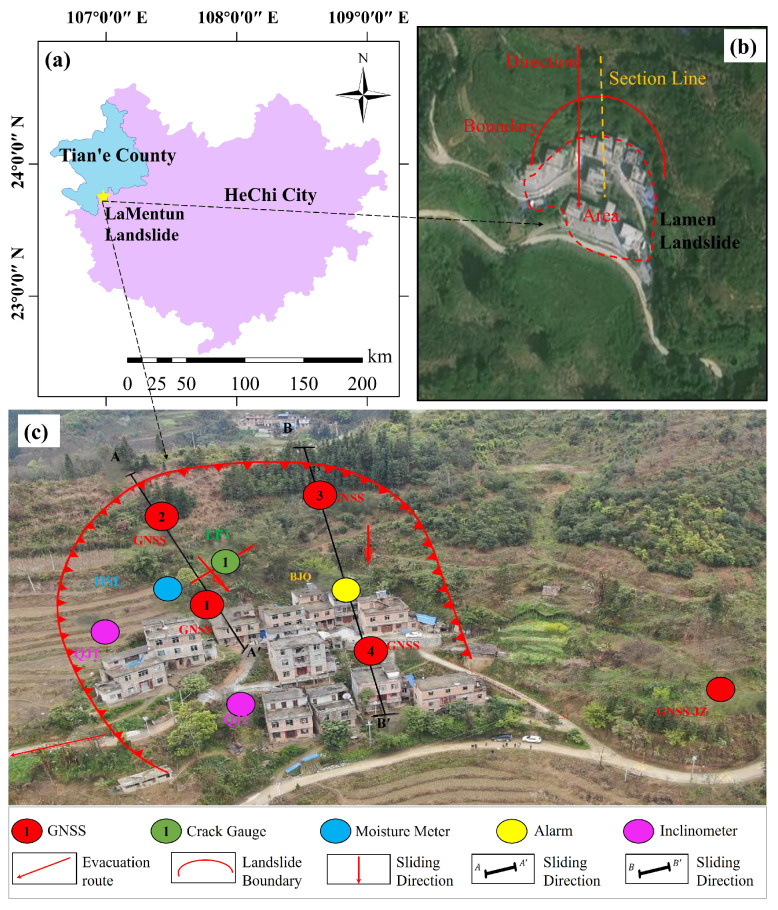
Layout of displacement and environmental monitoring instruments at the LaMenTun landslide. (**a**) Regional location map showing the LaMenTun landslide within Tian’e County, Hechi City, Guangxi, China; the five-pointed star marks the landslide site. (**b**) Satellite image of the landslide area, where the dashed boundary delineates the landslide extent, the arrow indicates the overall sliding direction, and the dashed line denotes the reference section line. (**c**) Field overview of the monitoring layout. Black dashed arrows are connector lines only, used to link corresponding locations/features between panels.

**Figure 6:**
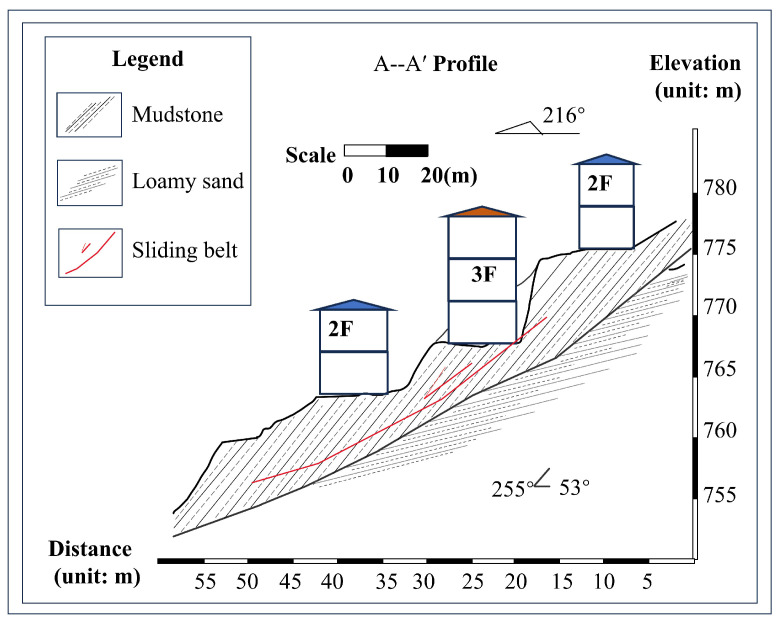
Spatial layout of the displacement and environmental monitoring instruments at the LaMenTun landslide site.

The LaMenTun landslide lies in Dangliang Village, Nazhi Township, Tian’e County, Guangxi. The landslide scar is semi-elliptical in plan within low-to-moderate local relief and partially encircles the local settlement (Figure 5). The source is at ∼800 m above sea level (a.s.l.) and the toe at ∼560 m, yielding ∼240 m of relief with a mean slip azimuth of $210^{{}^{\circ}}$. Terrace regrading and slope cutting further weaken the NE–SW-trending slope. The bedrock consists of muddy siltstone overlain by 0.8–2.8m of reddish-brown colluvial clay. The contact forms a soft-over-hard, dip-parallel sliding surface that weakens during intense rainfall (Figure 6). Historical failures in 2009 and 2011, together with active tensile cracks, indicate ongoing instability. The LaMenTun landslide monitoring network, comprising four GNSS stations, one reference station, one crack meter, a rain gauge, and soil moisture and soil temperature sensors, was established and commissioned on 30 March 2021 (Figure 5c).

This study compiles the multisource dataset collected by the network from 30 March 2021 to 28 June 2022, which includes daily GNSS displacement, rainfall, soil temperature, and volumetric water content. Outliers are filtered using physically informed thresholds. Soil temperature readings outside [−5,50] °C are removed. Sudden displacement shifts are flagged by a second derivative threshold (acceleration $>5\;\mathrm{mm}\;{\mathrm{day}}^{-2}$) and cross-validated with crack meter data. We aggregate all variables to daily means, and displacement and rainfall gaps shorter than three days are linearly interpolated. Soil variables are reconstructed with depth-aware cubic splines. Longer outages, accounting for <1% of timestamps, are masked and excluded from loss computation. No synthetic values beyond minimal interpolation are introduced, ensuring that occasional gaps do not affect forecast reliability.

The dataset reveals pronounced station-dependent deformation at LaMenTun (Figure 3). GPS03 shows the largest cumulative displacement (∼700 mm by mid-2025), with clear step-wise increments, including an abrupt ∼300 mm jump in mid-2022 and several smaller steps during 2023–2025. GPS01 undergoes slow, quasi-seasonal creep (∼5–65 mm) with modest accelerations and no sharp offsets. GPS04 records steady creep with a few moderate steps (total ∼100 mm). GPS02 is the least mobile (generally <30mm), with weak seasonality and short-period fluctuations. The LF01 crack meter widens in a step-wise manner, broadly synchronous with the main GNSS steps, especially in mid-2022 and mid-2023.

### 4.2. BaYiTun Landslide

The BaYiTun landslide is located southeast of BaYiTun Hamlet, Nandan County, Guangxi. It sits in a karst hill–canyon setting with ∼180 m local relief. Residual slope deposits of gravelly, clay-rich colluvium overlie Carboniferous tuff, forming a soft-over-hard interface that weakens during intense rainfall. Two N–S fractures and pervasive joints facilitate rapid groundwater routing (see [59] for details). The monitoring system includes one GNSS reference (JZ03), GPS01–GPS03 (GPS04 retired 4 August 2021), a co-located rain gauge (YL01), and soil moisture/temperature probes at 20–80 cm. Measurements at 06:00, 12:00, and 18:00 are aggregated to daily values. The dataset analyzed here spans 6 November 2021 to 28 June 2025. Data quality control, temporal aggregation, and gap handling follow the unified pipeline in Section 4.1.

**Figure 7:**
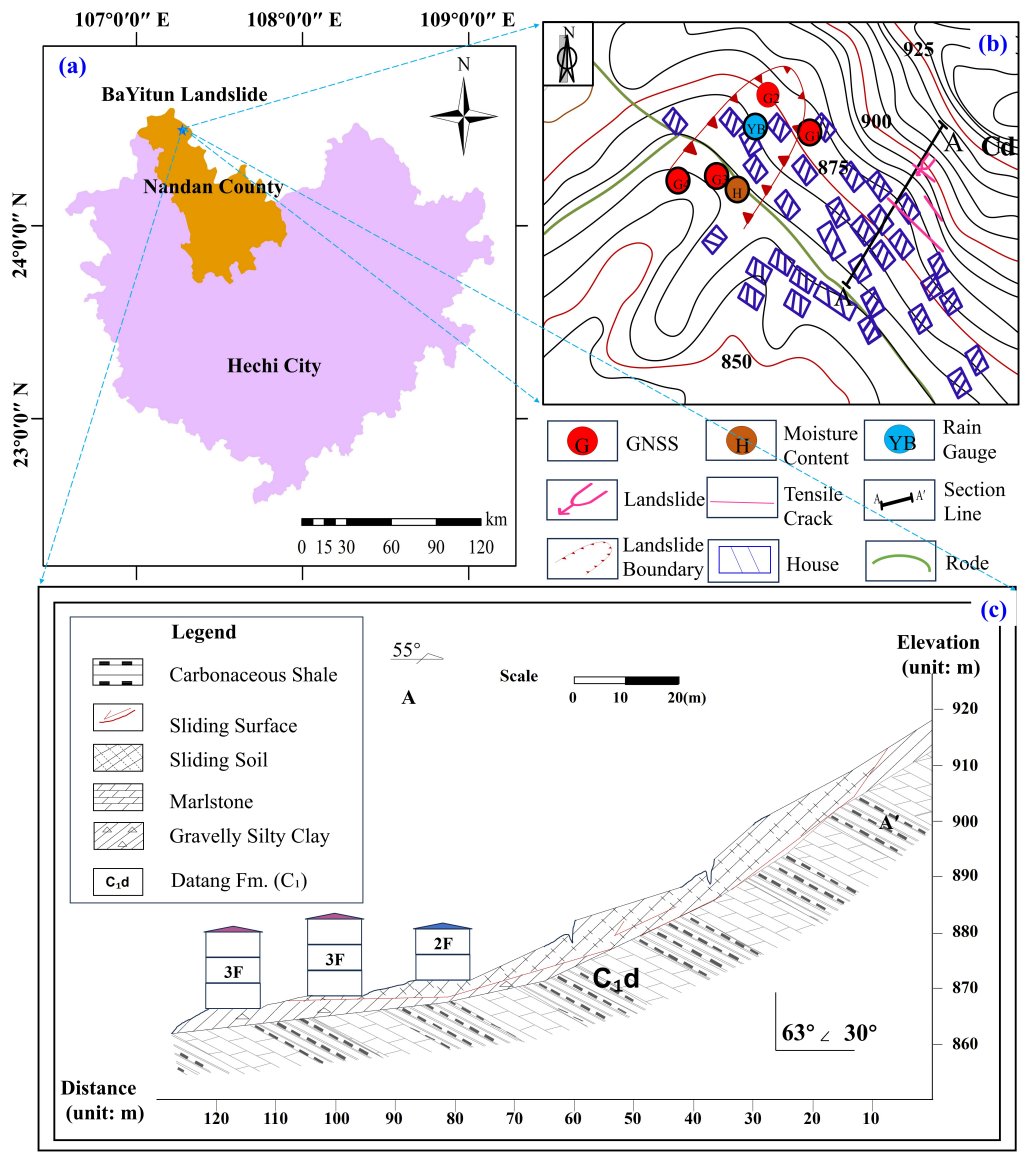
Layout of displacement and environmental monitoring instruments at the BaYiTun landslide. (**a**) Regional location in Nandan County, Hechi City, Guangxi, China; the five-pointed star marks the landslide site. (**b**) Plan view of the landslide showing instrument distribution and key features; symbol colors denote instrument types (see legend), and arrows/lines mark the landslide boundary/extent, tensile cracks, and the section line. (**c**) Geological profile along the section line in (**b**), showing stratigraphic units and the sliding surface/soil (see legend), with buildings for reference.

BaYiTun exhibits abrupt, multistage step-wise motion (Figure 4): GPS01 (up-slope) remains largely stable with cumulative displacement <60 mm; GPS02 (near the crest) shows gradual creep with episodic surges; GPS03 (mid-slope; “G3” in Figure 7c) displays 20–60 mm step-wise increments during wet spells (10-day rainfall ≳120 mm), consistent with excavation-induced toe weakening and a regolith–tuff contact that channels pore pressure-driven shear. Among the stations, GPS03 shows the highest variance and strongest non-stationarity.

## 5. Results

### 5.1. Experimental Environment

All experiments are run on a single CPU-only workstation to keep resource constraints identical across models. The host runs 64-bit Windows 11 (build 26,100) on an Intel Core i7-9700 (eight cores, 3.0 GHz) with 32 GB of RAM, and we use Python 3.11.7 (Anaconda) and PyTorch 2.2.1 (cpuonly) to perform our calculations. GPU acceleration is disabled (CUDA_VISIBLE_DEVICES = “”, torch.version.cuda = None, torch.backends.cudnn.enabled = False). To ensure reproducibility, we enable deterministic algorithms (torch.use_deterministic_algorithms(True)) and fixed random seeds for Python, NumPy, and PyTorch. Table 1 summarizes the key software and hardware components.

### 5.2. Performance Metrics

We evaluate model performance using four metrics: the mean absolute error (MAE), the root mean squared error (RMSE) [60], the coefficient of determination ($R^{2}$) [61], and the turning-point mean absolute error (${\mathrm{MAE}}_{\mathrm{turn}}$).

(39) $$\mathrm{MAE}=\frac{1}{n}\sum_{i=1}^{n}\left|{\hat{y}}_{i}-y_{i}\right|,$$

(40) $$\mathrm{RMSE}=\sqrt{\frac{1}{n}\sum_{i=1}^{n}{\left({\hat{y}}_{i}-y_{i}\right)}^{2}},$$

(41) $$R^{2}=1-\frac{\sum_{i=1}^{n}{\left(y_{i}-{\hat{y}}_{i}\right)}^{2}}{\sum_{i=1}^{n}{\left(y_{i}-\bar{y}\right)}^{2}},$$

The turning-point error is calculated as follows: Let $\Delta y_{i}=\left|y_{i}-y_{i-1}\right|$ for i=2,…,n. Define T as the index set of the top-p% samples (we use p=20) ranked by $\Delta y_{i}$ in the observed series. If needed, ties at the threshold are broken arbitrarily; the first sample is excluded from ranking). Then

(42) $${\mathrm{MAE}}_{\mathrm{turn}}=\frac{1}{\left|T\right|}\sum_{i\in T}\left|{\hat{y}}_{i}-y_{i}\right|.$$

where ${\hat{y}}_{i}$ denotes the prediction, $y_{i}$ the observation, $\bar{y}$ the mean of observations, and *n* the number of samples. ${\mathrm{MAE}}_{\mathrm{turn}}$ emphasizes accuracy at rapid transitions (large |Δy|), complementing aggregate metrics. In the main experiments, we define the turning-point error ${\mathrm{MAE}}_{\mathrm{turn}20}$ as the mean absolute error computed on the top 20% of samples ranked by |Δy|. This 20% threshold is chosen as a pragmatic compromise: it focuses the metric on large displacement changes that are most relevant for early warning while retaining enough samples to obtain stable statistics at each station.

### 5.3. Baseline Models and Experimental Protocol

#### 5.3.1. Baseline Models

We benchmark CRAFormer against four representative sequence models that span convolutional, recurrent, hybrid, and attention-based paradigms: a convolutional–recurrent hybrid (CNN–LSTM) [5], a gated recurrent unit (GRU) [62], a temporal convolutional network (TCN) [63], and a lightweight Transformer (LiteTransNet) [50]. These baselines (Table 2) are widely used for landslide displacement forecasting due to their ability to capture temporal dependencies and non-linearity across multiple scales.

We perform single-step forecasting (H=1) conditional on next-day rainfall. The exogenous rainfall at +1 day uses the co-located gauge’s next-day accumulation (oracle proxy), supplied equally to all models. At time *t*, the models predict $y_{t+1}$ from history $H_{t}$ and exogenous $R_{t+1}$; no other feature uses information beyond *t*. Inputs use a sliding window of length K=96 with stride 1. Accumulations terminate at *t*. Standardization is fit on the training split only and applied to validation/test (including $R_{t+1}$). Missing inputs within the window are imputed with training medians. Samples lacking $R_{t+1}$ are dropped.

Data are split chronologically: first 70% train, final 30% test. We grid-search hidden widths {32,64,128}, batch sizes {16,32,64}, and learning rates $\left\{5\times 10^{-3},10^{-3},5\times 10^{-4},10^{-4}\right\}$. Early stopping monitors the validation MAE (patience =20, max =100 epochs). All models use $L_{2}$ weight decay $10^{-4}$. Each configuration is run five times with seeds 2021–2025, and we report mean ± std on the test set. All runs are CPU-only with deterministic settings and pinned library versions. Multiday cumulative curves are rolling sums of one-day-ahead predictions and do not affect the H=1 protocol.

#### 5.3.2. Ablation Models

We quantify the contribution of each component using four ablations:

1. MLP (Correlational), a purely correlational baseline with no structure prior and no exogenous inputs.
2. MLP_Rain (Exogenous Evidence), which adds next-day rainfall $R_{t+1}$ as a leakage-free exogenous channel at prediction time while leaving the model unstructured to isolate the effect of evidence.
3. MLP + Granger (Linear CI), which serves as a weak causal baseline that uses linear Granger or partial correlation screening as a simple conditional independence filter for feature selection.
4. MLP_ DLCG (DBN-Style Structure Prior), which applies the learned DLCG and time-consistent structure masks that encode parents, ancestors, and collider visibility under non-anticipativity. The ICS exogenous tail is removed to test the structure prior alone.

Architectural details are summarized in Table 3.

### 5.4. Causal Graph and Role Mask Analysis

**Figure 8:**
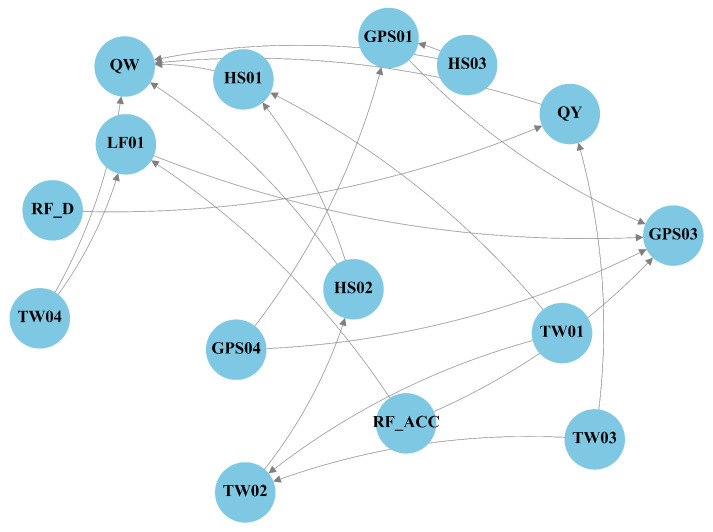
LaMenTun: DLCG-derived directed causal graph (DAG) over lagged variables. Edge orientations are determined by *v*-structures and Meek rules.

**Figure 9:**
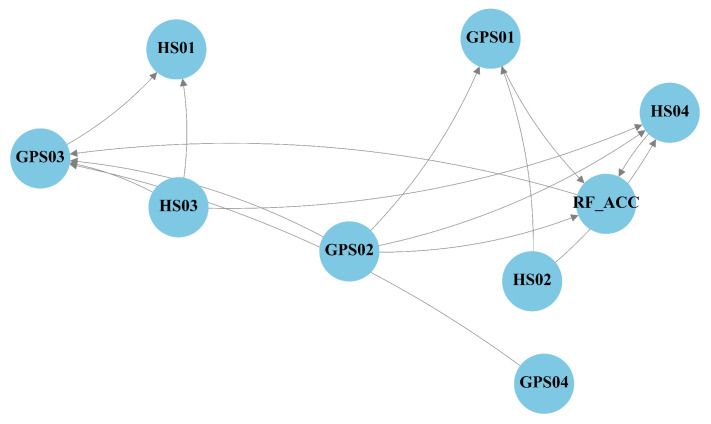
BaYiTun: DLCG-derived directed causal graph (DAG) over lagged variables. Edge orientations are determined by *v*-structures and Meek rules.

**Figure 10:**
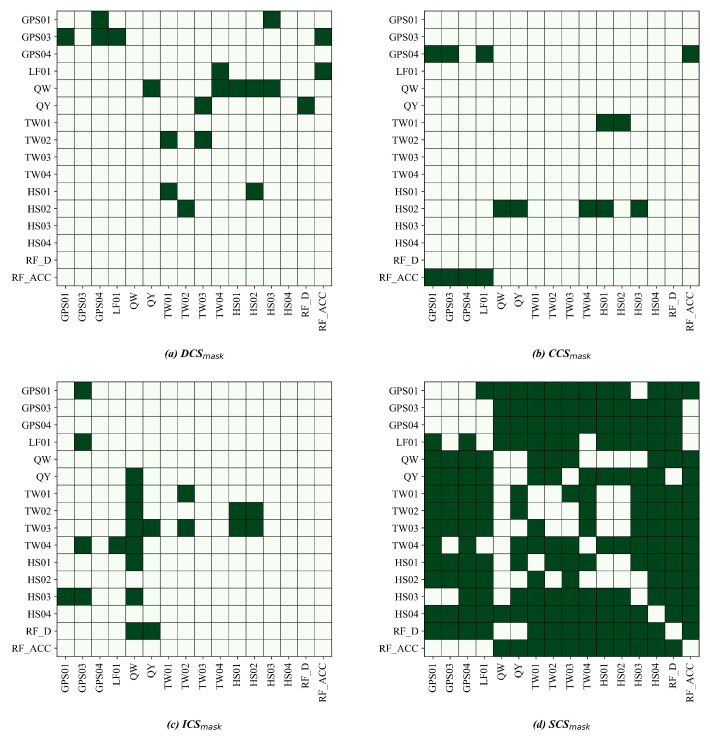
LaMenTun: role masks derived from the DLCG for the five-branch model. Panels (**a**–**d**) show ${\mathrm{DCS}}_{\mathrm{mask}}$, ${\mathrm{CCS}}_{\mathrm{mask}}$, ${\mathrm{ICS}}_{\mathrm{mask}}$, and ${\mathrm{SCS}}_{\mathrm{mask}}$, respectively. Dark green cells indicate allowed connections/visible entries (mask = 1), and light cells indicate masked entries (mask = 0).

**Figure 11:**
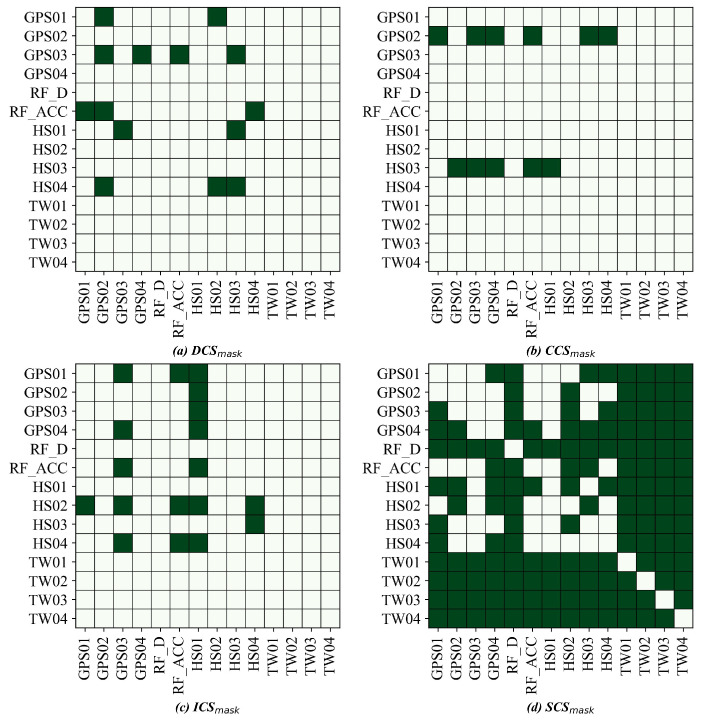
BaYiTun: role masks derived from the DLCG for the five-branch model. Panels (**a**–**d**) show ${\mathrm{DCS}}_{\mathrm{mask}}$, ${\mathrm{CCS}}_{\mathrm{mask}}$, ${\mathrm{ICS}}_{\mathrm{mask}}$, and ${\mathrm{SCS}}_{\mathrm{mask}}$, respectively. Dark green cells indicate allowed connections/visible entries (mask = 1), and light cells indicate masked entries (mask = 0).

Across both sites, the DLCGs summarize causal reachability (Figure 8 and Figure 9). We interpret these graphs as data-driven candidate pathways that guide the role masks and branch structure rather than an exhaustive set of physically verified mechanisms. Role masks convert these graphs into predictor visibility: DCSs retain one-hop parents; CCSs retain the collider and spouse sets; ICSs retain multistep mediators; and SCSs aggregate the remaining variables (Figure 10 and Figure 11).

At LaMenTun (Figure 8 and Figure 10), the DLCG is dense, and hydrometeorological influence arrives mainly through multistep paths. DCSs are sparse, ICSs are extensive, and many variables fall into the SCS category. Station-level patterns are consistent: GPS01 has few direct parents, and routes mostly influence via ICSs, with ES dominant during quiet periods. GPS03 retains key direct drivers in DCSs and adds soil thermal mediators in ICSs; the Top-2 gate often pairs ES or DCSs with ICSs during wet spells. GPS04 shows strong self-feedback and weak exogenous forcing, with a large SCS set. LF01 combines a small DCS with ICS mediators, consistent with episodic rainfall-sensitive widening.

At BaYiTun (Figure 9 and Figure 11), the DLCG is compact and centered on rainfall accumulation and shallow soil moisture. DCSs concentrate these short-lag parents, ICSs are small, and many temperature nodes are assigned to SCSs. Station-level patterns are consistent with this focus: GPS01 shows limited exogenous forcing and predominantly SCSs, consistent with stable behavior. GPS02 contains focused DCS parents from accumulation and shallow moisture, together with a few ICS mediators; short-lag hydrologic control strengthens during wet periods, whereas ES dominates otherwise. GPS03 shows the strongest inbound influence; DCS retain shallow moisture and accumulation parents, ICSs add a few hydrothermal mediators, and the Top-2 gate assigns higher weight to ICSs around displacement jumps.

### 5.5. Causal Relationship Analysis

#### 5.5.1. Diagnostics of Short-Lag Hydrologic Controls

In this section, we test whether the short-lag hydrologic pathways suggested by the causal graphs are supported by the data. Descriptive wavelet analysis indicates short-lag hydrometeorological influence at both sites, whereas predictive tests with BY-FDR control highlight site-specific mechanisms.

**Figure 12:**
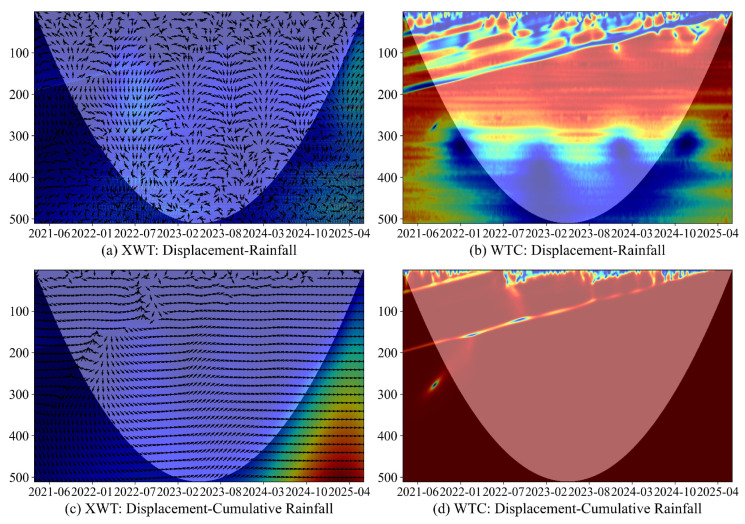
LaMenTun (GPS03): XWT/WTC between displacement and daily/cumulative rainfall. Colors denote magnitude (XWT: cross-wavelet power; WTC: coherence from 0 to 1), arrows indicate relative phase (lead/lag), and the shaded region marks the cone of influence.

At LaMenTun, station behavior is highly heterogeneous: GPS03 shows step-wise jumps superimposed on slow creep. LF01 widens in step-wise episodes that broadly track the main GNSS steps. Wavelet diagnostics for GPS03 (Figure 12) show short-period coherence after 2023 with rightward and upward phase arrows, indicating that rainfall precedes displacement by 1–5 days. For LF01, XWT and WTC reveal a seasonal band with soil temperature and patchy short-period coherence with cumulative rainfall that strengthens in late 2024 to 2025, again with drivers leading at short lags (Figure A5).

**Figure 13:**
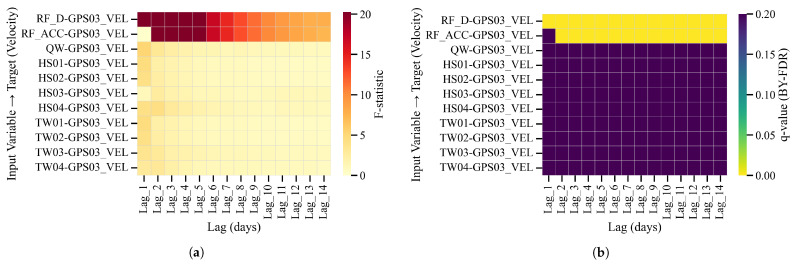
LaMenTun (GPS03): predictive Granger panels on velocity. (**a**) F-statistics over lags of 1–14 days; (**b**) BY–FDR *q*-values (bright = significant). No cell attains BY–FDR significance; short-lag F peaks do not survive multiplicity correction.

Predictive tests at LaMenTun are more conclusive: After BY-FDR correction across predictors and lags, the *q*-value panel shows a persistent low-*q* band for daily rainfall at short lags, whereas accumulated rainfall and most other variables are not significant (Figure 12 and Figure 13). Thus, near-lag daily rainfall is the primary predictor despite the broader candidates suggested by the wavelet analysis.

**Figure 14:**
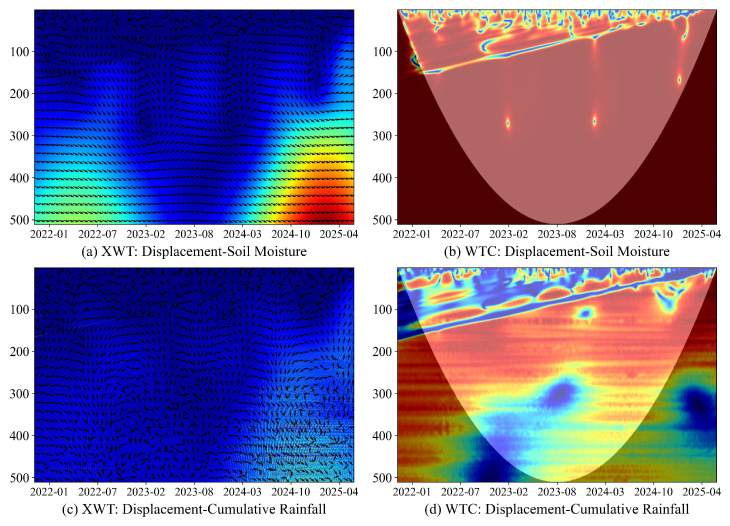
BaYiTun (GPS03): XWT/WTC between displacement and rainfall drivers. Colors denote magnitude (XWT: cross-wavelet power; WTC: coherence from 0 to 1), arrows indicate relative phase (lead/lag), and shading marks the cone of influence.

**Figure 15:**
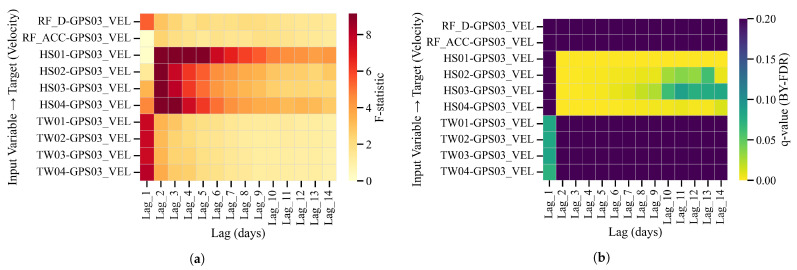
BaYiTun (GPS03): predictive Granger panels on velocity. (**a**) F-statistics over lags of 1–14 days; (**b**) BY–FDR *q*-values (bright = significant). A robust HS01–HS04 band appears at ∼2–10 days; rainfall rows are largely non-significant.

At BaYiTun, step-wise deformation is also observed. Descriptive wavelet coherence (WTC) and cross-wavelet transform (XWT) for GPS03 indicate compact short-period coherence with rainfall around displacement jumps (Figure 14), and predictive tests sharpen this picture: F-statistics peak for shallow soil moisture (HS01–HS04) at short-to-intermediate lags, and BY-FDR control yields a contiguous low-*q* band from 2 to 10 days. Daily rainfall and temperature are mostly not significant after correction (Figure 15).

From a mechanistic viewpoint, we distinguish DLCG pathways by confidence level. The highest-confidence edges are the short-lag links from daily rainfall (at LaMenTun) and shallow soil moisture HS01–HS04 (at BaYiTun) to displacement, together with ES lags. These are the only hydrologic drivers that receive consistent support from the wavelet and BY–FDR-controlled Granger diagnostics shown in Appendix B.3, and they align with the conceptual picture of infiltration, transient pore pressure build-up, and rate-dependent shear along the soft-over-hard contacts described in Section 4.1 and Section 4.2, as well as with the rainfall-stratified gate behavior described in Table 4. By contrast, DLCG edges involving soil temperature, deeper moisture probes, or collider/spouse relations among environmental variables are interpreted mainly as statistical proxies for shared seasonal forcing, the drainage state, or sensor co-location rather than as direct mechanical couplings. We therefore regard the former group as mechanistically interpretable and empirically supported candidates for short-lag driver–response pathways, whereas the latter are treated as statistical proxies that motivate their inclusion in the structural prior but are not interpreted as established physical couplings.

#### 5.5.2. Rainfall-Stratified Gate Behavior

**Figure 16:**
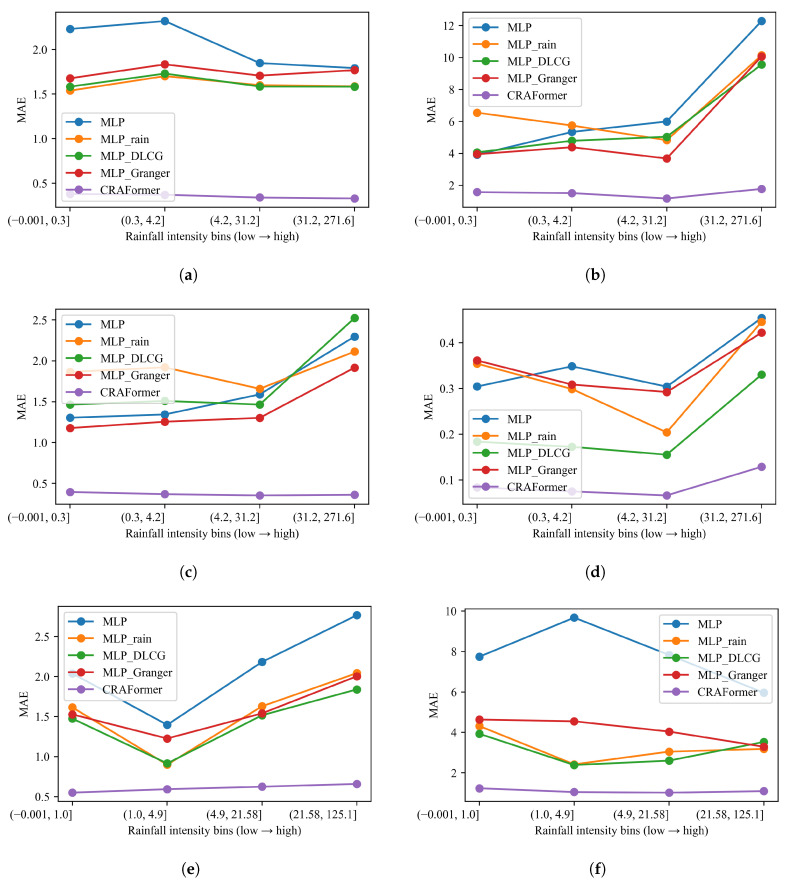
MAE vs. rainfall intensity (7-day accumulation) for six stations: (**a**) Lamen—GPS01; (**b**) Lamen—GPS03; (**c**) Lamen—GPS04; (**d**) Lamen—LF01; (**e**) Bayi—GPS02; (**f**) Bayi—GPS03.

**Figure 17:**
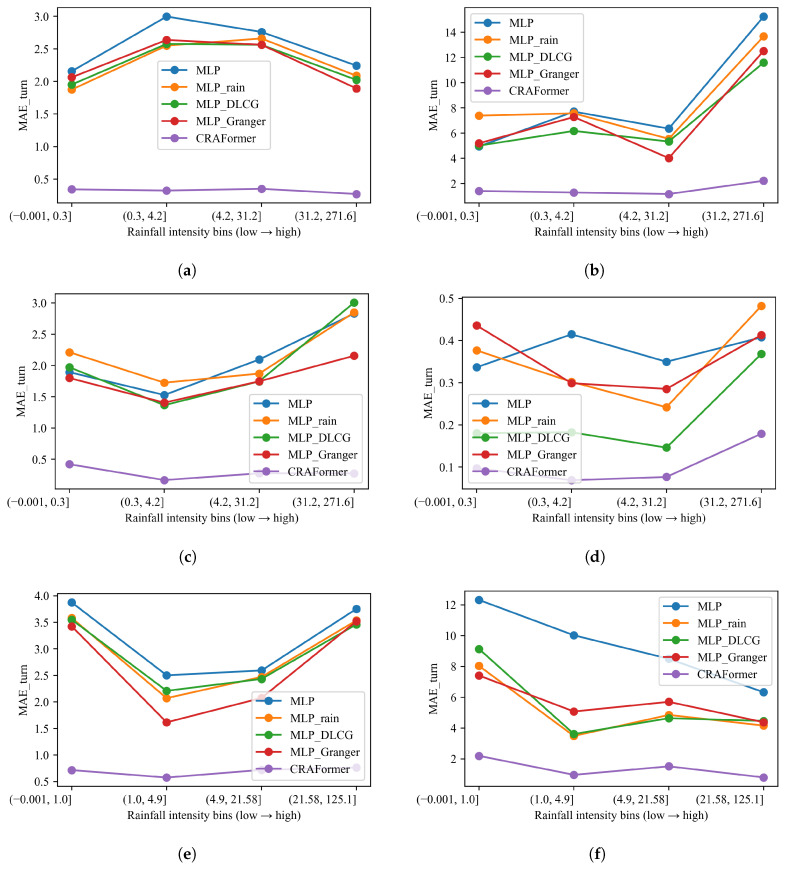
${\mathrm{MAE}}_{\mathrm{turn}}$ vs. rainfall intensity (top-20% |Δy|) using 7-day accumulation across six stations: (**a**) Lamen—GPS01; (**b**) Lamen—GPS03; (**c**) Lamen—GPS04; (**d**) Lamen—LF01; (**e**) Bayi—GPS02; (**f**) Bayi—GPS03.

We assess CRAFormer’s routing at the dataset level by aggregating pre-truncation gate probabilities ${\left\{\pi_{i,j}\right\}}_{j\in J}$ on the validation sets and stratifying them by 7-day accumulated rainfall $R_{7}$ (Dry/Moderate/Wet/Very Wet; same bins as Figure 16 and Figure 17). Table 4 reports for each station and bin the mean ES/DCS/ICS gate weights, the mean Top-2 mass $\pi_{i,(1)}+\pi_{i,(2)}$, and the mean entropy H(π).

Across all stations, ES dominates in Dry/Moderate regimes ($\pi_{\mathrm{ES}}\approx 0.6$, $\pi_{\mathrm{ICS}}\approx 0.05$–0.12), while ICS weights increase markedly with rainfall and become comparable to ES in Very Wet regimes (e.g., BaYiTun_gps03: $\pi_{\mathrm{ES}}=0.28$, $\pi_{\mathrm{ICS}}=0.37$). This shows that the model systematically up-weights ICSs under strong hydrometeorological forcing, rather than relying on anecdotal routing. The mean Top-2 mass is consistently high (about 0.79–0.88), and H(π) remains well below the five-way maximum log5≈1.61. Thus, the gate distribution is typically sharp and concentrates on, at most, two roles, so that, together with the Top-2 truncation (Section 3.3.1), CRAFormer does not average over all branches even when multiple roles are important, avoiding over-smoothing while adapting its routing to rainfall intensity.

### 5.6. Cumulative Displacement Prediction

#### 5.6.1. Comparative Evaluation of Baseline Models

**Figure 18:**
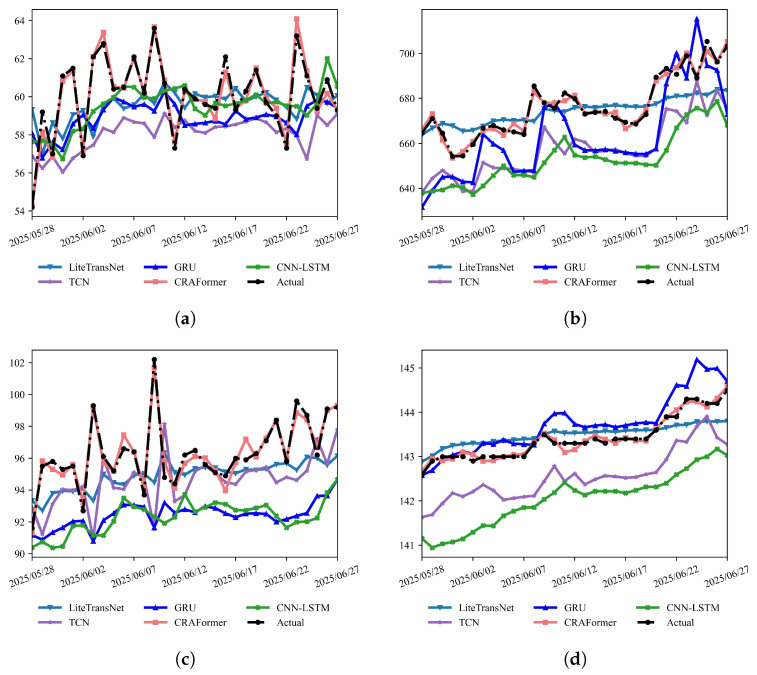
LaMenTun: comparison of baseline model prediction curves. Each panel shows daily predictions over the last month from LiteTransNet, GRU, CNN-LSTM, TCN, and CRAFormer against the observed series (black): (**a**) GPS01; (**b**) GPS03; (**c**) GPS04; (**d**) LF01.

**Figure 19:**
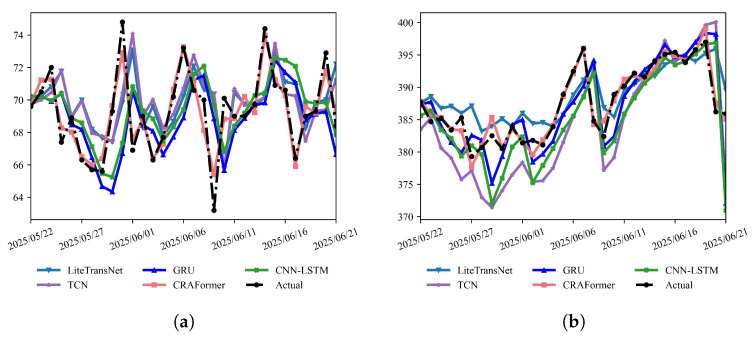
BaYiTun: baseline model prediction curves versus observed displacement over the last month. Models shown: LiteTransNet, GRU, CNN-LSTM, TCN, and CRAFormer. (**a**) GPS02; (**b**) GPS03.

For a comprehensive comparison, we evaluate six monitoring points: GPS01/GPS03/GPS04 and the crack meter LF01 at LaMenTun and GPS02/GPS03 at BaYiTun. We report the MAE, RMSE, $R^{2}$, and turning-sample error ${\mathrm{MAE}}_{\mathrm{turn}}$, defined as the mean absolute error over the top 20% of observed samples with the largest amplitude changes (treated as critical turning points). To assess whether the improvements of CRAFormer over the baselines are statistically significant, we form a time series of daily absolute errors for each station and model and apply a paired two-sided Wilcoxon signed-rank test between CRAFormer and the strongest non-proposed baseline, controlling multiplicity across stations and metrics with the Benjamini–Yekutieli FDR (BY–FDR) at a nominal level of 0.01. We also conduct a qualitative assessment based on daily predictions for the most recent month (Figure 18 and Figure 19).

As shown in Figure 18 and Figure 19, CRAFormer exhibits more stable phase alignment and more faithful amplitude reproduction than the baselines across both landslides. Taking LaMenTun as an example, GPS01, GPS03, and GPS04 display a compound pattern in late June, namely, an overall upward trend superimposed with high-frequency disturbances. CRAFormer remains in phase with the observations at both peaks and troughs and accurately captures the onset and termination of sharp rises. LiteTransNet fits the slowly increasing long-term trend reasonably well but shows a clear response lag and amplitude compression during the rapid upsurge on 21–27 June. Meanwhile, GRU tends to overshoot near extrema, and TCN commonly suffers from phase lag. For LF01, where crack displacement is dominated by mid-to-high-frequency, small-amplitude oscillations, CRAFormer responds more sensitively to short-period disturbances, with inflection points nearly coincident with the observations. By contrast, CNN–LSTM and TCN favor noise smoothing, partially weakening the depiction of peaks and valleys. At BaYiTun, GPS02 and GPS03 present the typical superposition of a low-frequency trend and high-frequency fluctuations. CRAFormer reliably identifies the turning points during the mid-month drop-and-rebound, whereas the other baselines exhibit varying degrees of amplitude compression and temporal misalignment in response to sudden disturbances (Figure 19).

The quantitative results are consistent with the observations in Table 5. Across all stations, CRAFormer attains the lowest MAE, RMSE, and turning-point errors ${\mathrm{MAE}}_{\mathrm{turn}10}$, ${\mathrm{MAE}}_{\mathrm{turn}20}$, and ${\mathrm{MAE}}_{\mathrm{turn}30}$, indicating that its advantage at rapid transitions is robust to the choice of percentile threshold. Focusing on the primary turning-point metric ${\mathrm{MAE}}_{\mathrm{turn}20}$, CRAFormer reduces the MAE and RMSE by approximately 60–70% and turning-point errors by about 70–80% relative to the best non-proposed baseline at both landslides, while maintaining a high $R^{2}$ of 0.97–0.99. At LaMenTun, for example, CRAFormer consistently lowers the ${\mathrm{MAE}}_{\mathrm{turn}20}$ across GPS01, GPS03, GPS04, and LF01, despite their mixed trend-plus-disturbance patterns. Similar relative gains are observed at BaYiTun for GPS02 and GPS03. Taken together, these findings show that CRAFormer robustly improves both overall errors and turning-point errors under varying geological settings and operational disturbances, and the same conclusion holds under the more stringent ${\mathrm{MAE}}_{\mathrm{turn}10}$ and more inclusive ${\mathrm{MAE}}_{\mathrm{turn}30}$ thresholds shown in Table 5. Paired Wilcoxon tests on daily absolute errors further indicate that, at all six stations, the improvements of CRAFormer over the strongest non-proposed baseline are statistically significant for the MAE and ${\mathrm{MAE}}_{\mathrm{turn}20}$ after BY–FDR adjustment at the 0.01 level.

#### 5.6.2. Ablation Analysis

**Figure 20:**
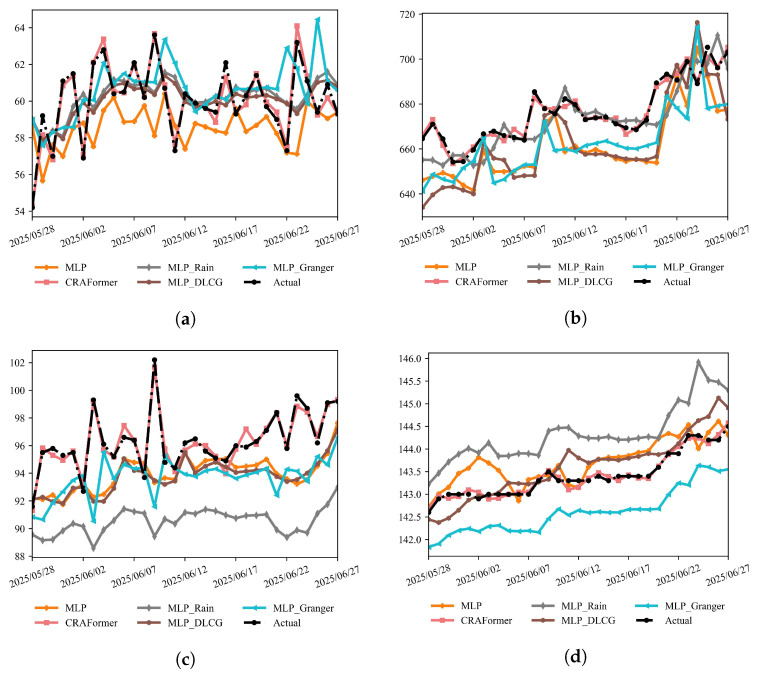
LaMenTun: ablation study prediction curves over the last month. Each panel compares MLP, MLP_Rain (exogenous evidence), MLP_ DLCG (DBN-style structure prior), MLP_Granger (linear CI screening), and CRAFormer against the observed series (black): (**a**) GPS01; (**b**) GPS03; (**c**) GPS04; (**d**) LF01.

**Figure 21:**
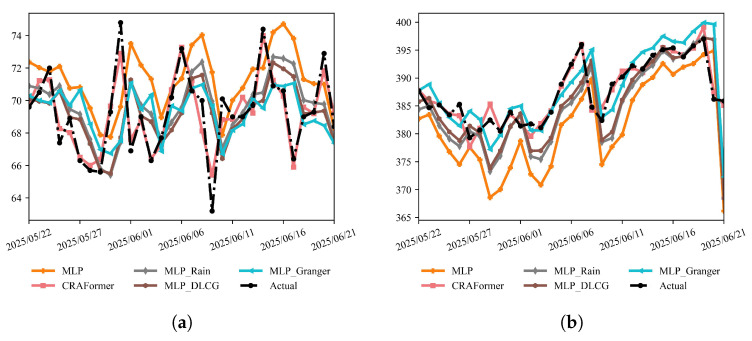
BaYiTun: ablation study prediction curves over the last month. Models shown are MLP, MLP_Rain, MLP_ DLCG, MLP_Granger, and CRAFormer versus the observed series (black): (**a**) GPS02; (**b**) GPS03.

To quantify the contribution of prior knowledge and variable screening, we evaluate four MLP-based variants, plain MLP, MLP_Rain (rainfall shifted by one day), MLP_DLCG (DBN-style dependency prior), and MLP_Granger (linear Granger causality screening), and compare them with CRAFormer on the same six stations (Figure 20 and Figure 21). We follow the baseline protocol and report the MAE, RMSE, $R^{2}$, and ${\mathrm{MAE}}_{\mathrm{turn}}$, computed over the top 20% of turning samples.

The overlays for the last month (Figure 20 and Figure 21) show that all MLP variants capture parts of the low-frequency trend but fail to track sharp transitions reliably. At LaMenTun (GPS01, GPS03, and GPS04), CRAFormer remains phase-coherent with the observations, reproducing the late-June surge and intermediate fluctuations, whereas MLP and MLP_Rain exhibit lag and amplitude compression around the surge and MLP_DLCG oversmooths peaks. MLP_Granger reduces spurious variability but still misplaces several turning points. For LF01, which is dominated by mid-to-high-frequency, small-amplitude oscillations, CRAFormer better tracks short cycles and inflection points, whereas the MLP-based models either attenuate extremes or introduce phase slippage. Similar patterns occur at BaYiTun: during the mid-month drop and rebound, CRAFormer aligns the timing and magnitude of peaks and troughs more closely, whereas the MLP variants display varying degrees of lag or overshoot.

These qualitative patterns align with the station-wise metrics in Table 6. Across all LaMenTun stations, CRAFormer reduces the MAE and RMSE by roughly 60–75% and lowers the ${\mathrm{MAE}}_{\mathrm{turn}20}$ by about 70–85% relative to the strongest MLP variants, while keeping $R^{2}$ in the 0.98–0.99 range. Similar margins are observed at BaYiTun, where CRAFormer consistently outperforms MLP, MLP_Rain, MLP_DLCG, and MLP_Granger on both overall errors and turning-point metrics. Importantly, CRAFormer attains the lowest turning-point errors at all three percentile levels (${\mathrm{MAE}}_{\mathrm{turn}10}$, ${\mathrm{MAE}}_{\mathrm{turn}20}$, and ${\mathrm{MAE}}_{\mathrm{turn}30}$), indicating that the gains are not an artifact of a particular threshold choice. Consistent with the baseline comparison, paired Wilcoxon tests on daily absolute errors show that CRAFormer also significantly outperforms the best-performing MLP variant on MAE and ${\mathrm{MAE}}_{\mathrm{turn}20}$ at all stations after BY–FDR correction at the 0.01 level.

These results indicate that the Rain(+1) variant MLP_rain, which appends next-day accumulated rainfall $R_{t+1}$ as an exogenous input, benefits trend-dominated regimes (e.g., GPS01) but degrades performance under non-linear or non-stationary rainfall–displacement couplings (e.g., GPS04, LF01), leading to lag and amplitude compression near sharp transitions. The DLCG stabilizes the MLP and offers modest gains under noise (LF01, GPS03), yet turning-point errors remain elevated because cross-scale interactions are not modeled explicitly. Granger screening prunes redundant drivers and can reduce variance (GPS03), but, owing to its linearity, it cannot correct phase shifts or capture abrupt non-linear changes, leaving timing mismatches at peaks and troughs. Overall, explicit exogenous cues and structural or linear priors provide targeted but insufficient improvements for mixed-trend-plus-disturbance regimes. By contrast, CRAFormer uses cross-scale attention and channel re-weighting to preserve long-term trends while enhancing responsiveness to short-term, high-frequency perturbations. This behavior is consistent with the station-wise reductions in the MAE, the RMSE, and especially the ${\mathrm{MAE}}_{\mathrm{turn}20}$, and the same pattern is observed at ${\mathrm{MAE}}_{\mathrm{turn}10}$ and ${\mathrm{MAE}}_{\mathrm{turn}30}$, as shown in Table 5 and Table 6.

#### 5.6.3. Sensitivity to Rainfall Intensity: Bin-Wise Assessment of Model Errors

Next, we stratify errors by 7-day accumulated rainfall (Figure 16). Across all six stations, CRAFormer attains the lowest MAE in every bin and shows weak sensitivity to rainfall intensity. Its curves remain nearly flat on GPS03, GPS04, and LF01, and rise only mildly in the heaviest rain bin on GPS01 and GPS02. By contrast, the MLP family degrades as rainfall increases. On LaMenTun—GPS01, MLP_Rain outperforms plain MLP in the low-to-moderate bins but spikes under the heaviest rain, indicating lag and amplitude compression around rain-driven surges. MLP_DLCG is steadier in the mid-range bins but deteriorates sharply at the highest intensity. MLP_Granger reduces variance but still remains well above CRAFormer. Similar trends hold on GPS03 and GPS04 and at BaYiTun (GPS02 and GPS03), where all MLP variants escalate under heavy rain but CRAFormer remains comparatively stable.

Applying the same stratification to the turning-sample error ${\mathrm{MAE}}_{\mathrm{turn}}$ (top 20% by |Δy|) yields a consistent picture (Figure 17). CRAFormer maintains uniformly low turning-point errors across bins, even in the heaviest rain bin, indicating strong phase alignment at rapid transitions. In contrast, the MLP variants exhibit pronounced sensitivity: MLP and MLP_Rain surge in the highest bin on GPS01, GPS02, and GPS03. MLP_DLCG reduces errors at moderate rainfall but cannot prevent sharp increases during extremes. MLP_Granger sometimes lowers variability at intermediate bins yet still misplaces turning points when rainfall intensifies. Taken together, the bin-wise analyses confirm that explicit exogenous cues or linear or structural priors yield limited resilience to hydrometeorological extremes, whereas CRAFormer sustains both trend accuracy and turning-point fidelity across rainfall regimes.

#### 5.6.4. Sensitivity of the ICS Branch to 24 h Rainfall Forecast Uncertainty

In CRAFormer, the ICS branch ingests the next-day rainfall $R_{t+1}$ via an exogenous tail token, which is set to the realized gauge accumulation in the main experiments. This represents an optimistic “oracle” setting, whereas real early-warning operations can only access numerical weather prediction (NWP) forecasts with random and systematic errors. To assess how such forecast-like uncertainty affects CRAFormer, we design a stress test in which the oracle rainfall is replaced by perturbed 24 h forecasts and evaluate the resulting change in predictive accuracy.

We use the metrics described in Section 5.2 (MAE, RMSE, $R^{2}$, and turning-point error ${\mathrm{MAE}}_{\mathrm{turn}}$ on the top 20% of samples ranked by |Δy|). Within each station, CRAFormer under oracle rainfall serves as the reference. For each NWP-like scenario, we report the relative changes as follows:

(43) $$\begin{array}{cc} \Delta \mathrm{MAE}(\%) & =100\times \frac{{\mathrm{MAE}}_{\mathrm{scenario}}-{\mathrm{MAE}}_{\mathrm{CRAFormer}}}{{\mathrm{MAE}}_{\mathrm{CRAFormer}}}, \end{array}$$

(44) $$\begin{array}{cc} \Delta {\mathrm{MAE}}_{\mathrm{turn}}\left(\%\right) & =100\times \frac{{\mathrm{MAE}}_{\mathrm{turn},\;\mathrm{scenario}}-{\mathrm{MAE}}_{\mathrm{turn},\;\mathrm{CRAFormer}}}{{\mathrm{MAE}}_{\mathrm{turn},\;\mathrm{CRAFormer}}}. \end{array}$$

where negative values indicate improvement over the oracle baseline.

To isolate the effect of rainfall forecast uncertainty on the ICS branch, we fix the displacement and driver histories for each station and re-evaluate CRAFormer on the last-month test subset under four rainfall scenarios: (i) *CRAFormer (oracle)*, using the realized gauge rainfall $R_{t+1}$; (ii) *NWP-mild*, representing a high-quality 24 h forecast with small bias and variance; (iii) *NWP-typical*, representing median 24 h forecast skill; and (iv) *NWP-poor*, representing degraded 24 h forecasts under convective or rapidly evolving conditions [64,65]. The NWP-like scenarios are constructed by injecting multiplicative bias and additive Gaussian noise into the oracle rainfall, following simple perturbation strategies used in statistical post-processing of NWP precipitation forecasts [66]. The perturbation parameters are chosen to be broadly consistent with reported 24 h QPF error ranges for subtropical monsoon regions. In all scenarios, only the exogenous next-day rainfall fed to the ICS tail token is perturbed; historical drivers and model parameters remain unchanged.

Table 7 confirms that oracle gauge rainfall is an optimistic reference and that 24 h forecast uncertainty affects CRAFormer in a site-dependent way. At LaMenTun—GPS01, GPS03, and GPS04, replacing oracle $R_{t+1}$ with NWP-like inputs typically increases the MAE by O(10–50%) and reduces the $R^{2}$ to about 0.91–0.97, indicating that forecast noise injected through the ICS tail can weaken trend fitting when daily rainfall plays a strong causal role. The crack meter LF01 is an extreme case: in the NWP-poor scenario, the MAE more than triples and ${\mathrm{MAE}}_{\mathrm{turn}}$ increases by almost an order of magnitude, suggesting that highly biased or noisy forecasts may trigger spurious ICS responses at high-frequency, noise-dominated sensors. In such regimes, CRAFormer should be combined with forecast quality screening or explicit down-weighting of the ICS gate. In contrast, BaYiTun stations exhibit weaker sensitivity: at BaYiTun—GPS02, all NWP scenarios slightly reduce the MAE and strongly reduce the ${\mathrm{MAE}}_{\mathrm{turn}}$ while maintaining a high $R^{2}$, consistent with shallow soil moisture rather than daily rainfall being the primary short-lag driver. At BaYiTun—GPS03, forecast perturbations mainly affect the smooth background component, while the timing of sharp displacement changes remains comparatively robust, reflecting the dominant role of short-lag hydrological controls encoded in the historical drivers.

## 6. Discussion

This study focuses on cross-site differences in the predictability of the rainfall–displacement relationship. We employ a unified diagnostic framework comprising WTC/XWT, predictive Granger testing with BY-FDR correction, and falsification via temporal and rainfall permutations to test our modeling hypotheses, and we cross-validate them using attributions from the model’s gated routing. We specifically examine whether the evidence supports short-lag hydrologic control and use this to explain CRAFormer’s behavior across different deformation regimes.

**Figure A3:**
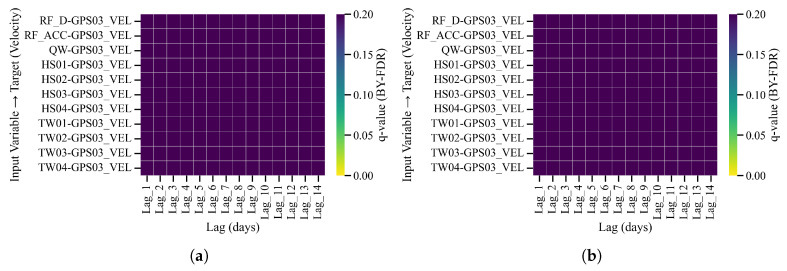
LaMenTun (GPS03): BY–FDR *q*-value panels for misalignment settings. No cell attains significance under any setting, consistent with Figure 13. (**a**) Temporal misalignment (+7 d); (**b**) spatial mismatch proxy (rainfall block permutation).

At LaMenTun (GPS03 as an example), although the wavelet plots show short-period in-phase bands, none of the lags within 1–14 days remains significant after BY-FDR correction, and the falsification tests are also null (Figure A3), indicating a lack of predictive signal (Figure 12). Consistently, the gating weights favor ES/DCSs over time, and ICSs are only briefly activated during wet spells. For LF01, the narrow window (approximately 6–8 days) weakens under the spatial mismatch test, which further supports the conclusion that there is no robust short-lag driver at this site (Figure A6 and Figure A8).

**Figure A4:**
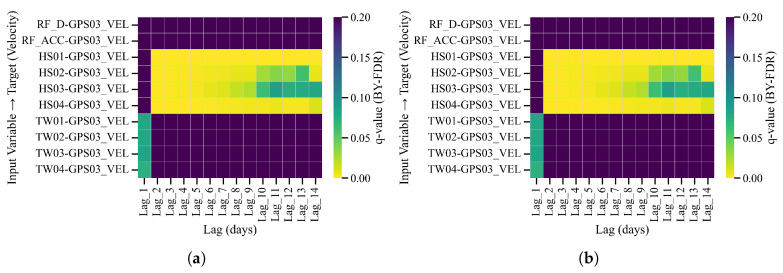
BaYiTun (GPS03): BY–FDR *q*-value panels under misalignment settings. A robust HS01–HS04 band persists around 2–10 d; rainfall is non-significant throughout, in agreement with Figure 15. (**a**) Temporal misalignment (+7 d); (**b**) spatial mismatch proxy (rainfall block permutation).

**Figure A5:**
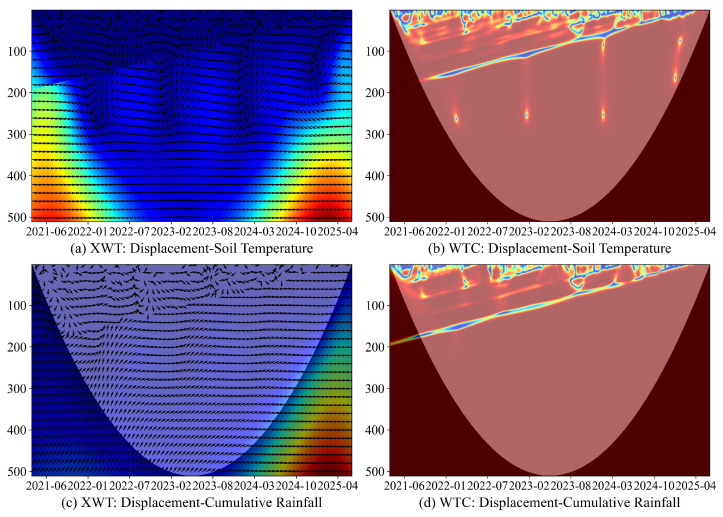
LaMenTun (LF01): XWT/WTC between displacement and rainfall drivers. Colors denote magnitude (XWT: cross-wavelet power; WTC: coherence from 0 to 1), arrows indicate relative phase (lead/lag), and shading marks the cone of influence.

**Figure A6:**
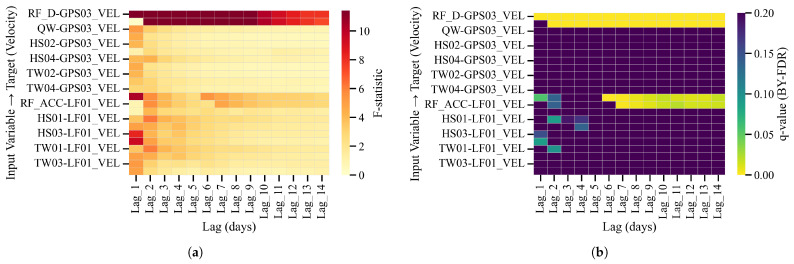
LaMenTun (LF01): predictive Granger results for displacement velocity (first differences). Short-lag F peaks appear widely, but, after BY–FDR, only RF_Acc_ exhibits a narrow significant window at ∼6–8 d. (**a**) Granger strength (F-statistic); (**b**) BY–FDR-corrected *q*-values.

**Figure A8:**
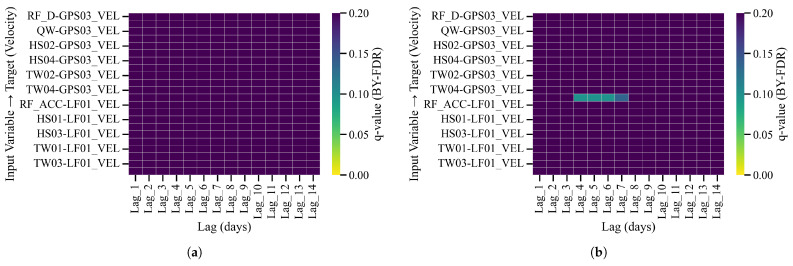
LaMenTun (LF01): BY–FDR *q*-value panels for misalignment settings. No cell attains significance under any setting, consistent with Figure 13. (**a**) Temporal misalignment (+7 d); (**b**) spatial mismatch proxy (rainfall block permutation).

**Figure A9:**
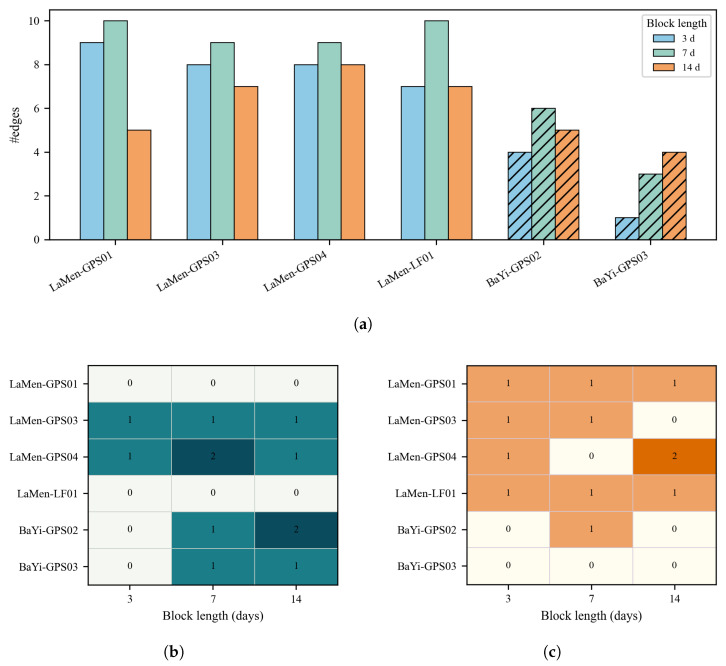
Sensitivity of theDLCG structure to the stationary bootstrap block length. (**a**) Total number of edges |E| for block lengths b∈{3,7,14} days; bar colors (and hatching where applicable) indicate different block lengths. (**b**) Size of the direct-cause set |DCS|; cell color intensity encodes the magnitude (lighter = smaller, darker = larger), and the overlaid numbers give the exact values. (**c**) Size of the indirect-cause set |ICS|; cell color intensity encodes the magnitude (lighter = smaller, darker = larger), and the overlaid numbers give the exact values.

At BaYiTun (GPS03), shallow soil moisture (HS01–HS04) exhibits a stable BY-FDR-significant band over roughly 2–10 days, and this signal retains strength under permutation-based falsification (Figure A4). During step events, WTC/XWT also reveals a hydrology-leads displacement-lags temporal pattern (Figure 14). In line with this, the model assigns more Top-2 routing mass to ICSs during wet periods while maintaining background constraints from ES/DCSs, and it substantially reduces timing bias and magnitude errors near turning points (Figure 10 and Figure 11).

Taken together, these results substantiate the causal, role-masked design of CRAFormer. Across both landslides, CRAFormer preserves phase and amplitude at turning points and step onsets (Figure 18, Figure 19, Figure 20 and Figure 21), yielding large, station-consistent reductions in MAE, RMSE, and especially ${\mathrm{MAE}}_{\mathrm{turn}}$ (typically 56–86%; Table 5 and Table 6). At LaMenTun, the gains arise from robust tracking of background drift when ES/DCS processes dominate. At BaYiTun, the Top-2 gate up-weights ICSs during jump episodes while remaining coupled to ES/DCSs, thereby curbing timing bias and overshoot. These patterns are consistent with the rainfall-stratified analyses indicating CRAFormer’s low sensitivity under heavy rain (Figure 16 and Figure 17). In our experiments, the rainfall-triggered monotonicity prior sharpens timing and magnitude at wet turning points without introducing visible artifacts during post-event relaxation, consistent with its design as a soft, regime-specific regularizer rather than a hard global constraint.

Beyond predictive skill, the diagnostics also inform the working assumptions behind the DLCG described in Section 3.1. Wavelet coherence and Granger panels (Section 5.5) indicate that only a small set of short-lag hydrologic drivers has substantial predictive power across seasons and step-like episodes. In practice, near-lag rainfall at LaMenTun and shallow soil moisture at BaYiTun carry most of the short-lag signal once multiplicity is controlled. The pronounced non-stationarity in displacement levels is largely absorbed by ES self-lags, which is consistent with dominant ES gate weights in Dry and Moderate rainfall bins (Table 4). These patterns support the working assumption that short-lag driver–response dependencies are approximately stable over time, even though the raw displacement series is not.

From a predictive standpoint, the ablation study in Section 5.6.2 and the rainfall-stratified error curves show that removing DLCG-based role masks mainly increases MAE and ${\mathrm{MAE}}_{\mathrm{turn}}$ without producing unstable or clearly spurious behavior under heavy rainfall. This suggests that moderate departures from the DLCG assumptions primarily reduce the incremental benefit of the structural prior rather than materially degrading forecast robustness. Together with the role stability check in Appendix A.2, this indicates that sampling variability in the DLCG structure has limited impact on the effective set of causal roles used by the Top-2 gate. At the level of individual edges, we interpret only the short-lag rainfall links, the shallow soil–moisture links, and the ES self-lags as the best-supported mechanistic candidates, and treat the remaining DLCG connections mainly as a statistical structure in the prior rather than as confirmed physical couplings (Section 5.5).

CRAFormer was designed for practicality, with CPU-only training, single-head attention, and small branches without prior signal decomposition which simplify maintenance and curb error propagation. When archived forecasts are unavailable, we use realized next-day rainfall as a proxy. Reported scores therefore characterize performance under accurate rainfall inputs, and real-time deployments will scale with forecast quality. Although we have not yet reported explicit hit, miss, or false-alarm rates, the systematic reductions in MAE, RMSE, and especially ${\mathrm{MAE}}_{\mathrm{turn}}$ suggest fewer and smaller timing errors near rapid displacement surges, which is directly relevant for threshold-based early-warning schemes. These improvements document robustness across two contrasting deformation regimes within a shared humid, subtropical setting in Guangxi, but extension to landslides in other climatic or geomorphological environments will require additional validation and possibly modest adaptation of the causal driver set.

Our co-variates include daily and cumulative rainfall, soil temperature, and soil moisture. Pore pressure, water level, and inter-station geometry were not available, so we trained CRAFormer per station, which may under-represent cross-slope propagation and shared hydrologic forcing. Incorporating pore pressure and water level records as short-lag drivers, together with inter-station geometry and shared hydrologic pathways in a spatially coupled DLCG (e.g., a graph over GNSS and hydrologic sensors), should sharpen the timing and magnitude of predicted accelerations, reduce reliance on displacement self-history, and better distinguish genuine slope responses from sensor noise, particularly for deep-seated or reservoir-affected landslides. In future work, we will extend CRAFormer from single-station to multistation configurations that learn a joint DLCG over sensor networks and ingest real-time spatial information from dense GNSS arrays and, where available, InSAR-derived deformation fields.

## 7. Conclusions

This work introduced CRAFormer, a compact causality- and physics-aware predictor that replaces signal decomposition with DLCG-based role masks and a Top-2 gating mechanism for fusion. The ICS branch ingests next-day rainfall through a leakage-free tail with a stop-gradient (detach) operation, non-negative mixing, and a monotonicity prior, yielding forecast-compatible yet non-anticipative behavior. Under oracle conditioning, where the same observed $R_{t+1}$ is provided to all models, CRAFormer consistently outperformed the baselines across two rainfall-triggered landslides in Guangxi that share a humid, subtropical, monsoon-dominated climate but differ markedly in their deformation regimes. It achieved the largest gains in high-rainfall bins and at turning points, reducing timing bias as well as overshoot and undershoot. Ablations showed incremental gains from MLP_Rain and MLP_DLCG, yet CRAFormer remained superior. Causal diagnostics aligned with the learned routing: LaMenTun showed no short-lag drivers that were significant under the BY–FDR procedure, whereas BaYiTun exhibited a robust 2–10-day shallow moisture lag band. The model is lightweight (single-head attention, CPU-friendly) and interpretable via sample-wise gates. The present evidence is restricted to two rainfall-triggered landslides in Guangxi and is further limited by the absence of pore pressure and water level data, the lack of explicit spatial coupling, the use of oracle conditioning, and an evaluation focused on continuous regression and turning-point errors rather than explicit hit, miss, and false-alarm rates under operational thresholds. We expect that incorporating pore pressure and water level observations as non-anticipative exogenous drivers, learning spatially coupled DLCGs over sensor networks, and using operational forecast products together with threshold-based and probabilistic early-warning metrics will strengthen the physical interpretability of the inferred pathways, further reduce timing errors near rapid accelerations, and broaden applicability to landslides with stronger hydrogeologic control and more complex internal kinematics.

## Figures and Tables

**Table 1:** Experimental environment summary.

Component	Specification
Operating system	Windows 11 (Build 26,100)
CPU/memory	Intel Core i7-9700 (8 cores, 3.0 GHz)/32 GB RAM
GPU	None (CPU-only)
CUDA/cuDNN	disabled
Python env	Python 3.11.7 (Anaconda)
PyTorch	2.2.1 (cpuonly)
Core libraries	NumPy, Pandas, Matplotlib (v3.8.4), scikit-learn, statsmodels
Determinism	torch.use_deterministic_algorithms(True); fixed seeds

**Table 2:** Architectural configurations of baseline models.

Model	Structure Summary	Activation
TCN	Three TemporalBlocks, where each block has two Conv1D layers (kernel =16; dilation =1,2,4); channel width =H; residual connections	ReLU
CNN–LSTM	Conv stack: Conv1D(D→H) (kernel =16) → Conv1D(H→H) (kernel =32), max-pool; then LSTM (hidden =H) over pooled sequence; head: fc H→1	ReLU (Conv), tanh/sigmoid (LSTM)
GRU	Three-layer GRU (input dimension $=n_{\mathrm{features}}$, hidden units =H); output head: fc H→1	ReLU (head)
LiteTransNet	2 encoder + 2 decoder layers, where each encoder: 1×4-head attention; each decoder: 2×4-head attention; FFN: H→4H→H; head: fc H→1	ReLU

**Table 3:** Architectural configurations of ablation variants and the full model, where *K* is the look-back window, *D* is the number of features, *H* is the MLP width, and *d* is the Transformer width.

Model	Structure Summary	Key Settings
MLP	Three-layer MLP on flattened window: fc1 $R^{K\;\times \;D}\;\to \;H$, fc2 H→H/2, fc3 H/2→1; no masks, no exogenous input.	H∈{32,64,128}; ReLU; dropout 0; $L_{2}\;=\;10^{-4}$
MLP_Rain	As MLP; append leakage-free $R_{t+1}$ at prediction time as an exogenous scalar; no structure masks.	H∈{32,64,128}; ReLU; dropout 0; $L_{2}\;=\;10^{-4}$
MLP + Granger	As MLP; inputs pre-screened by linear Granger/partial-corr CI (per-lag, BY–FDR); no learned masks.	H∈{32,64,128}; ReLU; BY–FDR α=0.05; $L_{2}\;=\;10^{-4}$
MLP_DLCG	As MLP; apply DLCG time-consistent visibility masks on lag-unrolled graph (parents/ancestors/colliders; non-anticipativity); no ICS tail.	H∈{32,64,128}; ReLU; DLCG: HSIC/KCI+BY–FDR, bootstrap; $L_{2}\;=\;10^{-4}$
CRAFormer	Five role branches (ES/DCS/CCS/ICS/SCS) with single-head causal self-attention (*d*) and lite Transformer cell; ICS uses exogenous $R_{t+1}$ tail (leakage-free, non-negative readout, monotonic regularization); Top-2 context-aware gating; convex fusion.	d∈{32,64,128}; GELU/ReLU; dropout 0.1; $L_{2}=10^{-4}$; $\lambda_{\mathrm{ent}}\in \left[10^{-3},10^{-2}\right]$; $\lambda_{\mathrm{scs}}\in \left[10^{-3},10^{-2}\right]$; $\lambda_{\mathrm{mono}}\in \left[10^{-3},10^{-1}\right]$

Note: Inputs are standardized on the training split; default K=96, stride 1. Optimizer Adam; lr $\in \{5\times 10^{-3},10^{-3},5\times 10^{-4},10^{-4}\}$; early stopping on val-MAE (patience 20, max 100 epochs). Rain(+1) is used strictly as exogenous evidence (no gradients). DLCG discovery excludes future nodes and enforces forward-time edges; masks implement *d*-separation and non-anticipativity.

**Table 4:** Rainfall-stratified mean pre-truncation gate weights for ES, DCSs, and ICSs, mean Top-2 gate mass, and mean gate entropy H(π) at LaMenTun and BaYiTun stations.

Station	Bin	ES Mean π	DCS Mean π	ICS Mean π	Mean Top-2 Mass	Mean H(π)	*N* Samples
LaMenTun_gps01	Dry	0.66	0.18	0.05	0.87	0.64	115
	Moderate	0.58	0.19	0.10	0.85	0.71	110
	Wet	0.45	0.21	0.20	0.82	0.82	135
	Very Wet	0.34	0.21	0.29	0.80	0.93	58
LaMenTun_gps03	Dry	0.62	0.20	0.07	0.86	0.68	108
	Moderate	0.53	0.21	0.14	0.84	0.76	102
	Wet	0.40	0.22	0.26	0.81	0.87	142
	Very Wet	0.30	0.22	0.35	0.79	0.98	64
LaMenTun_gps04	Dry	0.68	0.17	0.05	0.88	0.62	120
	Moderate	0.60	0.18	0.09	0.86	0.69	118
	Wet	0.49	0.20	0.17	0.83	0.80	130
	Very Wet	0.38	0.21	0.24	0.81	0.90	55
LaMenTun_lf01	Dry	0.63	0.18	0.06	0.86	0.66	96
	Moderate	0.55	0.20	0.12	0.84	0.74	92
	Wet	0.43	0.21	0.22	0.82	0.84	104
	Very Wet	0.33	0.22	0.31	0.80	0.96	49
BaYiTun_gps02	Dry	0.65	0.17	0.05	0.87	0.63	103
	Moderate	0.57	0.19	0.10	0.85	0.71	123
	Wet	0.46	0.21	0.19	0.82	0.82	113
	Very Wet	0.35	0.22	0.27	0.80	0.93	113
BaYiTun_gps03	Dry	0.61	0.20	0.07	0.86	0.67	90
	Moderate	0.52	0.20	0.15	0.84	0.77	86
	Wet	0.38	0.22	0.27	0.81	0.88	100
	Very Wet	0.28	0.23	0.37	0.79	1.00	47

“ES mean π”, “DCS mean π”, and “ICS mean π” are the average pre-truncation gate weights for the ES, DCS, and ICS branches, respectively, within each 7-day rainfall regime. “Mean Top-2 mass” is the average sum of the two largest gate probabilities $\pi_{i,(1)}+\pi_{i,(2)}$. H(π) denotes the mean entropy of the five-way gate distribution; *N* is the number of validation samples falling into each bin.

**Table 5:** Model performance on LaMenTun and BaYiTun stations with turning-point errors.

Station	Model	MAE	RMSE	$R^{2}$	${\mathrm{MAE}}_{\mathrm{turn}10}$	${\mathrm{MAE}}_{\mathrm{turn}20}$	${\mathrm{MAE}}_{\mathrm{turn}30}$
LaMenTun_gps01	TCN	1.799	2.255	0.735	3.219	2.740	2.423
	GRU	1.592	2.038	0.762	3.522	2.899	2.496
	CNN_LSTM	1.681	2.139	0.731	3.300	2.680	2.253
	LiteTransNet	1.656	2.092	0.971	3.516	3.002	2.613
	CRAFormer	**0.359**	**0.456**	**0.977**	**0.403**	**0.379**	**0.356**
LaMenTun_gps03	TCN	6.136	9.078	0.958	13.891	12.138	10.260
	GRU	6.554	9.220	0.953	12.860	11.308	9.535
	CNN_LSTM	6.196	8.213	0.938	9.728	8.930	8.104
	LiteTransNet	5.045	6.883	**0.998**	10.339	7.863	7.423
	CRAFormer	**1.525**	**1.947**	0.996	**1.741**	**1.660**	**1.596**
LaMenTun_gps04	TCN	1.561	2.101	0.904	3.955	3.033	2.605
	GRU	1.664	2.249	0.876	3.693	2.847	2.293
	CNN_LSTM	1.629	2.218	0.867	3.590	2.883	2.371
	LiteTransNet	1.411	1.845	**0.989**	3.371	2.621	2.233
	CRAFormer	**0.374**	**0.471**	0.983	**0.391**	**0.391**	**0.382**
LaMenTun_lf01	TCN	0.359	0.569	0.978	0.773	0.556	0.425
	GRU	0.226	0.455	0.974	0.568	0.397	0.328
	CNN_LSTM	0.429	0.700	0.946	0.967	0.709	0.545
	LiteTransNet	0.342	0.454	**0.995**	0.690	0.544	0.459
	CRAFormer	**0.090**	**0.144**	**0.995**	**0.128**	**0.113**	**0.093**
BaYiTun_gps02	TCN	1.530	1.994	0.815	3.944	3.323	2.787
	GRU	1.740	2.257	0.888	4.127	3.210	2.616
	CNN_LSTM	1.458	1.943	0.821	3.957	3.115	2.531
	LiteTransNet	1.623	2.122	**0.993**	3.904	3.174	2.740
	CRAFormer	**0.594**	**0.765**	0.973	**0.820**	**0.721**	**0.647**
BaYiTun_gps03	TCN	5.253	7.164	0.825	7.436	5.864	5.607
	GRU	3.115	4.997	0.929	7.396	5.253	4.553
	CNN_LSTM	3.541	5.352	0.896	7.368	5.531	4.841
	LiteTransNet	3.504	5.454	**0.995**	8.315	6.079	5.121
	CRAFormer	**1.131**	**1.588**	0.991	**1.792**	**1.399**	**1.342**

**Table 6:** Ablation results on LaMenTun and BaYiTun stations with turning-point errors.

Station	Model	MAE	RMSE	$R^{2}$	${\mathrm{MAE}}_{\mathrm{turn}10}$	${\mathrm{MAE}}_{\mathrm{turn}20}$	${\mathrm{MAE}}_{\mathrm{turn}30}$
LaMenTun_gps01	MLP	2.084	2.612	0.230	4.109	3.439	2.836
	MLP_rain	1.589	2.051	0.525	3.468	2.835	2.418
	MLP_DLCG	1.609	2.066	0.518	3.457	2.847	2.403
	MLP_Granger	1.729	2.231	0.438	3.639	3.050	2.466
	CRAFormer	**0.359**	**0.456**	**0.977**	**0.403**	**0.379**	**0.356**
LaMenTun_gps03	MLP	6.293	8.541	0.917	11.249	9.713	8.891
	MLP_rain	6.773	8.714	0.914	11.450	9.987	8.670
	MLP_DLCG	5.514	7.551	0.935	9.425	8.662	7.865
	MLP_Granger	5.214	7.978	0.928	10.837	9.250	7.920
	CRAFormer	**1.525**	**1.947**	**0.996**	**1.741**	**1.660**	**1.596**
LaMenTun_gps04	MLP	1.565	2.083	0.675	3.563	2.698	2.279
	MLP_rain	1.883	2.330	0.593	3.271	2.561	2.375
	MLP_DLCG	1.681	2.172	0.647	3.377	2.622	2.297
	MLP_Granger	1.360	1.826	0.750	3.372	2.701	2.334
	CRAFormer	**0.374**	**0.471**	**0.983**	**0.391**	**0.391**	**0.382**
LaMenTun_lf01	MLP	0.343	0.495	0.938	0.509	0.427	0.381
	MLP_rain	0.332	0.495	0.938	0.631	0.508	0.449
	MLP_DLCG	0.205	0.423	0.955	0.605	0.422	0.339
	MLP_Granger	0.350	0.520	0.932	0.685	0.554	0.474
	CRAFormer	**0.090**	**0.144**	**0.995**	**0.128**	**0.113**	**0.093**
BaYiTun_gps02	MLP	2.084	2.649	0.667	3.805	3.305	2.663
	MLP_rain	1.560	2.061	0.799	3.782	3.078	2.493
	MLP_DLCG	1.443	1.936	0.822	3.761	3.046	2.470
	MLP_Granger	1.566	2.077	0.795	3.742	2.868	2.350
	CRAFormer	**0.594**	**0.765**	**0.972**	**0.820**	**0.721**	**0.647**
BaYiTun_gps03	MLP	7.768	9.296	0.708	11.209	9.306	8.632
	MLP_rain	3.462	5.226	0.908	7.071	5.211	4.682
	MLP_DLCG	3.277	5.138	0.911	7.497	5.537	4.753
	MLP_Granger	4.215	6.014	0.878	7.753	5.689	4.974
	CRAFormer	**1.131**	**1.588**	**0.991**	**1.792**	**1.399**	**1.342**

**Table 7:** CRAFormer under oracle and NWP-like 24 h rainfall scenarios at six stations.

Station	Scenario	MAE	RMSE	$R^{2}$	${\mathrm{MAE}}_{\mathrm{turn}}$	ΔMAE (%)	Δ${\mathrm{MAE}}_{\mathrm{turn}}$ (%)
lamen_gps01	CRAFormer	0.359	0.456	0.977	0.458	0.0	0.0
	NWP-mild	0.439	0.528	0.954	0.433	22.3	−5.5
	NWP-typical	0.478	0.593	0.912	0.430	33.1	−6.1
	NWP-poor	0.401	0.519	0.932	0.361	11.7	−21.2
lamen_gps03	CRAFormer	1.525	1.947	0.996	1.778	0.0	0.0
	NWP-mild	2.225	2.619	0.963	2.630	45.9	47.9
	NWP-typical	2.052	2.511	0.966	2.861	34.6	60.9
	NWP-poor	1.666	2.123	0.976	1.380	9.2	−22.4
lamen_gps04	CRAFormer	0.374	0.471	0.983	0.506	0.0	0.0
	NWP-mild	0.418	0.534	0.936	0.322	11.8	−36.4
	NWP-typical	0.580	0.697	0.891	0.583	55.1	15.2
	NWP-poor	0.415	0.558	0.930	0.383	11.0	−24.3
lamen_lf01	CRAFormer	0.090	0.144	0.995	0.043	0.0	0.0
	NWP-mild	0.081	0.156	0.952	0.091	-10.0	111.6
	NWP-typical	0.120	0.156	0.895	0.070	33.3	62.8
	NWP-poor	0.287	0.391	0.339	0.294	218.9	583.7
bayi_gps02	CRAFormer	0.594	0.765	0.973	1.222	0.0	0.0
	NWP-mild	0.540	0.810	0.960	0.396	−9.1	−67.6
	NWP-typical	0.584	0.817	0.924	0.506	−1.7	−58.6
	NWP-poor	0.565	0.827	0.920	0.248	−4.9	−79.7
bayi_gps03	CRAFormer	1.131	1.588	0.991	0.865	0.0	0.0
	NWP-mild	1.661	1.870	0.974	0.488	46.9	−43.6
	NWP-typical	1.461	1.585	0.988	0.476	29.2	−45.0
	NWP-poor	1.645	1.827	0.977	0.777	45.4	−10.2

**Figure A1:**
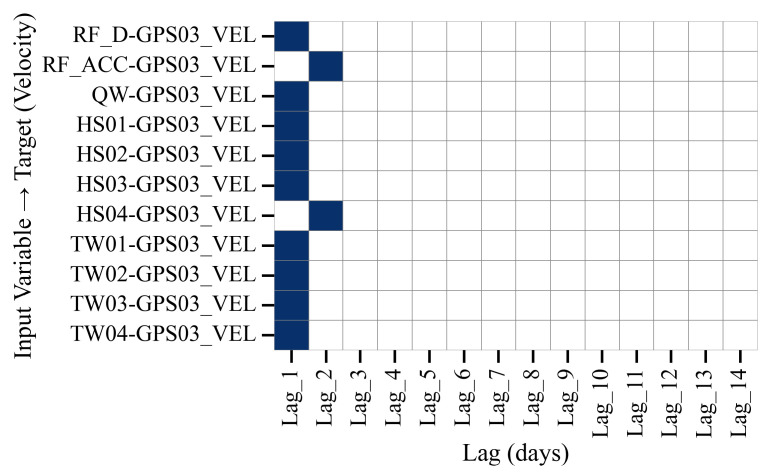
LaMenTun (GPS03): best-lag map ($L^{*}$ = earliest lag with the minimal *q*-value). Dark-blue cells mark the selected $L^{*}$ for each input–target pair; white cells indicate non-selected lags (not $L^{*}$). Entries concentrate at 1–2 d; however, none of the associated cells is BY–FDR-significant in Figure 13, so $L^{*}$ is descriptive rather than evidence of a robust causal lead–lag relation.

**Figure A2:**
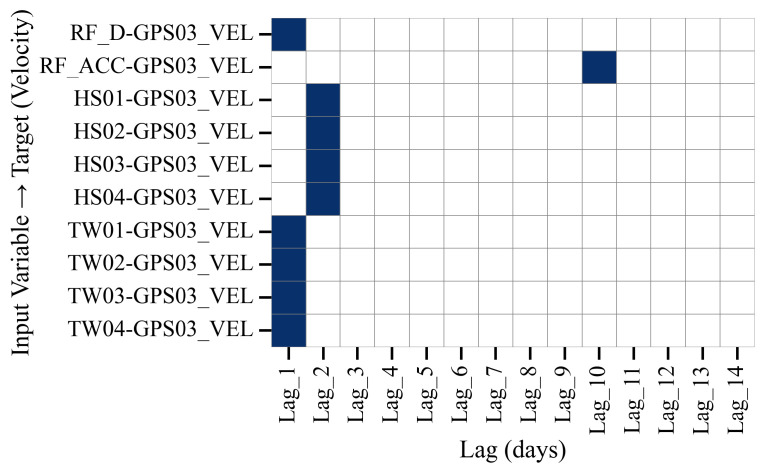
BaYiTun (GPS03):best-lag map ($L^{*}$ = earliest lag with the minimal *q*-value). Dark-blue cells mark the selected $L^{*}$ for each input–target pair; white cells indicate non-selected lags (not $L^{*}$). Shallow soil moisture (HS01–HS04) concentrates at $L^{*}\;\approx$ 2–3 d and RF_Acc_ near ∼10 d, agreeing with the BY–FDR-significant bands in Figure 15; RF_D_ and temperature yield $L^{*}\;\approx$ 1 d but are largely non-significant after correction.

**Figure A7:**
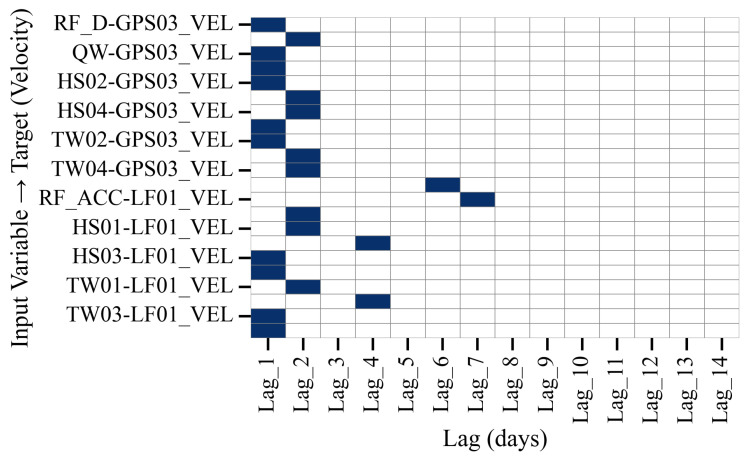
LaMenTun (LF01): best-lag map ($L^{*}$ = earliest lag with the minimal *q*-value). Dark-blue cells mark the selected $L^{*}$ for each input–target pair; white cells indicate non-selected lags (not $L^{*}$). Many entries fall at 1–2 d but are largely non-significant after BY–FDR; RF_Acc_ yields $L^{*}\approx 6$–7 d, consistent with Figure A6b.

## Data Availability

The datasets presented in this article are not readily available because they were provided by government departments and contain sensitive geospatial information. Requests to access the datasets should be directed to the first author.

## Appendix A

Scope: This appendix complements the main text by detailing the DLCG procedure and its hyperparameters.

### Appendix A.1. DLCG Hyperparameters and Selection Thresholds

We learn the DLCG on historical variables only; future rainfall nodes within the prediction horizon are excluded to avoid anticipative edges and are later handled as exogenous inputs in the ICS tail (see Section 3.3.1). Unless otherwise noted, we use the following procedure: To account for serial dependence, we apply a stationary bootstrap with *B* resamples and an expected block length of 7 days. For each resample, we run a constraint-based structure learner with HSIC/KCI as the primary conditional independence (CI) test (Gaussian kernel with median heuristic bandwidth), compute *p*-values by block permutation, and control false discoveries within each conditioning order using BY–FDR at level α=0.05. For low-order conditioning sets (|Z|≤2) that pass linearity and normality diagnostics, we fall back to the Gaussian partial correlation (Fisher–*z*) CI test. The pruned skeleton is then oriented via *v*-structures and Meek rules under hard temporal constraints.

Across bootstrap replications, we record forward and reverse directional selection rates for each ordered pair (u,v) and compute Clopper–Pearson confidence bounds for these binomial proportions. Let $L_{\to }$ and $U_{\leftarrow }$ denote the lower and upper Clopper–Pearson bounds for the forward (u→v) and reverse (v→u) directions, respectively, and apply the directional selection rule in Equation (4) to obtain the aggregated DLCG. To assess the robustness of this rule, we vary $\left(L_{\to },\Delta \right)$ over {(0.55,0.25),(0.60,0.30),(0.65,0.35)} at a representative station (BaYiTun—GPS03) and summarize the resulting aggregated DLCG and role masks in Table A1. The total edge count and the size of the ICS set change only mildly, while the DCS set for the displacement node is invariant (Jaccard index =1 for all configurations). In particular, short-lag rainfall and near-surface soil moisture drivers remain in the same direct- or indirect-cause roles, indicating that the causal structure used by CRAFormer is stable under moderate perturbations of the directional thresholds.

**Table A1:** Sensitivity of the aggregated DLCG and role masks under different directional selection thresholds $\left(L_{\to },\Delta \right)$ at the LaMenTun and BaYiTun stations.

Station	Config	Edges	DCS	ICS	Jacc (DCS)	Jacc (ICS)
LaMenTun_gps01	$L_{\to }=0.55,\;\Delta =0.25$	7	1	0	1.0	0.0
	$L_{\to }=0.60,\;\Delta =0.30$	7	1	1	1.0	1.0
	$L_{\to }=0.65,\;\Delta =0.35$	6	0	0	0.0	0.0
LaMenTun_gps03	$L_{\to }=0.55,\;\Delta =0.25$	7	0	1	0.0	0.0
	$L_{\to }=0.60,\;\Delta =0.30$	7	1	1	1.0	1.0
	$L_{\to }=0.65,\;\Delta =0.35$	6	1	0	1.0	0.0
LaMenTun_gps04	$L_{\to }=0.55,\;\Delta =0.25$	7	0	0	0.0	1.0
	$L_{\to }=0.60,\;\Delta =0.30$	7	1	0	1.0	1.0
	$L_{\to }=0.65,\;\Delta =0.35$	6	1	0	1.0	1.0
LaMenTun_lf01	$L_{\to }=0.55,\;\Delta =0.25$	7	0	0	1.0	0.0
	$L_{\to }=0.60,\;\Delta =0.30$	7	0	1	1.0	1.0
	$L_{\to }=0.65,\;\Delta =0.35$	6	0	0	1.0	0.0
BaYiTun_gps02	$L_{\to }=0.55,\;\Delta =0.25$	3	0	0	0.0	0.0
	$L_{\to }=0.60,\;\Delta =0.30$	3	1	1	1.0	1.0
	$L_{\to }=0.65,\;\Delta =0.35$	2	0	0	0.0	0.0
BaYiTun_gps03	$L_{\to }=0.55,\;\Delta =0.25$	3	1	0	1.0	0.0
	$L_{\to }=0.60,\;\Delta =0.30$	3	1	1	1.0	1.0
	$L_{\to }=0.65,\;\Delta =0.35$	2	1	1	1.0	1.0

“edges” is the total number of directed edges in the aggregated DLCG. “DCS” and “ICS” are the sizes of the direct-cause and indirect-cause sets for the corresponding displacement node. Jaccard indices are computed with respect to the reference configuration (L_→_, Δ) = (0.60, 0.30) at each station.

### Appendix A.2. Role Stability Check for DCS/ICS Masks

We recompute the DLCG on $B^{\prime }$ stationary bootstrap subsets at two representative stations (LaMenTun_gps03 and BaYiTun_gps03) and track the role assignments of short-lag hydrologic drivers for the displacement node. Table A2 reports, for each candidate lag, the proportion of runs in which it is assigned to DCSs, ICSs, or SCSs.

**Table A2:** Stability of DCS/ICS/SCS assignments for short-lag hydrologic drivers of displacement under stationary bootstrap resampling. Entries are the proportions of runs in which each lag appears in the corresponding role.

Station	Driver Lag	DCS	ICS	SCS
LaMenTun_gps03	rain (t−1)	0.92	0.08	0.00
LaMenTun_gps03	rain (t−2)	0.05	0.81	0.14
BaYiTun_gps03	HS01 (t−3)	0.88	0.12	0.00
BaYiTun_gps03	HS01 (t−5)	0.07	0.76	0.17

### Appendix A.3. DLCG Hyperparameter Summary Table

Table A3 lists all tunable (and fixed) hyperparameters used in the DLCG procedure described in Section 3.1. Unless otherwise stated, these values are held constant across all experiments.

**Table A3:** Hyperparameters for the DLCG. The CI selection rule uses two fixed thresholds (see Section 3.1).

Parameter	Description	Value/Range
*L*	Look-back window (lags)	24–48 (task-specific)
*B*	# stationary bootstrap resamples	≥20
*block_len*	Bootstrap block length (days)	7
$L_{\mathrm{col}}$	Collider–spouse mining depth	2–3
α	BY–FDR level per order	0.05
$E^{+},\;E^{-}$	Soft/hard prior edge sets	Domain-specific
γ	Tie-break exponent for $E^{+}$	0.5
$\left(\lambda^{+},\;\lambda^{-}\right)$	Soft prior weights	(0.2, 1.0)
CI rule	Edge retention thresholds	$L_{\to }\;\geq \;0.60$, $L_{\to }-U_{\leftarrow }\;\geq \;0.30$

## Appendix B. Negative Controls and Best-Lag Maps

Scope: We summarize best-lag ($L^{*}$) maps and report falsification and negative controls for both sites under four settings: aligned inputs, temporal shifts of +3 and +7 days, and a spatial proxy based on rainfall block permutation. Unless otherwise noted, targets use first-differenced displacement (velocity), input lags span 1 to 14 days, and all *q*-values are BY-FDR-corrected.

### Appendix B.1. Best-Lag Summary Maps (BY–FDR)

For each input→target pair, we take $L^{*}$, the earliest lag attaining the minimal *q*-value within the 1–14 day range.

### Appendix B.2. Falsification/Negative Control Panels (BY–FDR q-Values)

Panels show BY–FDR *q*-values for aligned inputs, +3/+7 day temporal shifts, and a rainfall block permutation spatial proxy.

### Appendix B.3. LaMenTun (LF01): Predictive Granger Panels, Best-Lag Map, and Falsification

Figure A6 summarizes predictive Granger tests for LaMenTun (LF01) using first-differenced displacement (velocity) as the target and input lags of 1 to 14 days. Raw F-statistics show mild short-lag peaks for several inputs (e.g., at 1 to 2 days), but, after BY-FDR control across all predictors, a *narrow* significant window remains, primarily for accumulated rainfall (${\mathrm{RF}}_{\mathrm{Acc}}$) around approximately 6 to 8 days. Most other predictors are not significant after multiplicity correction.

To compactly characterize the lag structure, we construct a best-lag map that selects, for each input→target pair, the earliest lag $L^{*}$ achieving the minimum *q*-value (Figure A7). At LF01, $L^{*}$ values concentrate at 1 to 2 days for a subset of variables, but these cells are *not* significant under the BY-FDR correction in Figure A6b and should therefore be regarded as descriptive only. In contrast, ${\mathrm{RF}}_{\mathrm{Acc}}$ achieves $L^{*}$ at approximately 6 to 7 days and aligns with the FDR-significant trough, suggesting a modest hydrologic memory at intermediate lags that is specific to accumulated rainfall at this site.

We further evaluate a falsification setting using a spatial mismatch proxy based on rainfall block permutation. As shown in Figure A8, the low-*q* window for ${\mathrm{RF}}_{\mathrm{Acc}}$ is attenuated under the proxy, and no new significant bands emerge. This suggests that the predictive signal is sensitive to proper spatial alignment of rainfall drivers and argues against spurious coupling between rainfall and displacement signals.

### Appendix B.4. Sensitivity to the Stationary Bootstrap Block Length

In the main experiments, we set the expected block length of the stationary bootstrap (and block permutation) to 7 days. This choice roughly matches the decorrelation time of daily rainfall and displacement at our sites, and the typical duration of storm-driven pore pressure responses, so it preserves short-term serial dependence without making the effective sample size too small.

To assess robustness, we re-run the DLCG for all six station–target pairs with block lengths b∈{3,7,14} days and summarize the total number of edges |E| and the sizes of the direct-cause and indirect-cause sets, |DCS| and |ICS| (Figure A9). Across sites, |E| varies by at most 2–3 edges and the role sizes change by at most one node when moving away from 7 days. The only visible difference is that BaYiTun—GPS02/03 gains or loses a single shallow soil moisture parent, while the remaining structure is unchanged. Overall, the key hydrological drivers and the DCS/ICS masks are stable, and the 7-day block length used in the main text is therefore a reasonable and robust choice.
